# A comprehensive draft genome sequence for lupin (*Lupinus angustifolius*), an emerging health food: insights into plant–microbe interactions and legume evolution

**DOI:** 10.1111/pbi.12615

**Published:** 2016-09-23

**Authors:** James K. Hane, Yao Ming, Lars G. Kamphuis, Matthew N. Nelson, Gagan Garg, Craig A. Atkins, Philipp E. Bayer, Armando Bravo, Scott Bringans, Steven Cannon, David Edwards, Rhonda Foley, Ling‐ling Gao, Maria J. Harrison, Wei Huang, Bhavna Hurgobin, Sean Li, Cheng‐Wu Liu, Annette McGrath, Grant Morahan, Jeremy Murray, James Weller, Jianbo Jian, Karam B. Singh

**Affiliations:** ^1^ CSIRO Agriculture Wembley WA Australia; ^2^ Department of Environment and Agriculture CCDM Bioinformatics Centre for Crop and Disease Management Curtin University Bentley WA Australia; ^3^ Curtin Institute for Computation Curtin University Bentley WA Australia; ^4^ Department of Plant and Animal Genome Research Beijing Genome Institute Shenzhen China; ^5^ UWA Institute of Agriculture University of Western Australia Crawley WA Australia; ^6^ School of Plant Biology University of Western Australia Crawley WA Australia; ^7^ Boyce Thompson Institute for Plant Research Ithaca NY USA; ^8^ Proteomics International Nedlands WA Australia; ^9^ USDA‐ARS Corn Insects and Crop Genetics Research Unit Crop Genome Informatics Lab Iowa State University Ames IA USA; ^10^ Department of Agronomy Iowa State University Ames IA USA; ^11^ University of Queensland Brisbane Qld Australia; ^12^ Data61 CSIRO Canberra ACT Australia; ^13^ John Innes Centre Norwich Research Park Norfolk UK; ^14^ Centre for Diabetes Research University of Western Australia Crawley WA Australia; ^15^ School of Biological Sciences University of Tasmania Hobart TAS Australia; ^16^ Present address: Royal Botanic Gardens Kew Natural Capital and Plant Health Ardingly RH17 6TN UK

**Keywords:** Legume comparative genomics, synteny, whole‐genome assembly, flowering time genes, polyploidy, Genistoids

## Abstract

Lupins are important grain legume crops that form a critical part of sustainable farming systems, reducing fertilizer use and providing disease breaks. It has a basal phylogenetic position relative to other crop and model legumes and a high speciation rate. Narrow‐leafed lupin (NLL;* Lupinus angustifolius* L.) is gaining popularity as a health food, which is high in protein and dietary fibre but low in starch and gluten‐free. We report the draft genome assembly (609 Mb) of NLL cultivar Tanjil, which has captured >98% of the gene content, sequences of additional lines and a dense genetic map. Lupins are unique among legumes and differ from most other land plants in that they do not form mycorrhizal associations. Remarkably, we find that NLL has lost all mycorrhiza‐specific genes, but has retained genes commonly required for mycorrhization and nodulation. In addition, the genome also provided candidate genes for key disease resistance and domestication traits. We also find evidence of a whole‐genome triplication at around 25 million years ago in the genistoid lineage leading to *Lupinus*. Our results will support detailed studies of legume evolution and accelerate lupin breeding programmes.

## Introduction

Lupins are grain legumes that form an integral part of sustainable farming systems and have been an important part of the human diet for thousands of years (Gladstones, 1970). Planted in rotation with cereal crops, lupins reduce the need for nitrogenous fertilizer, provide valuable disease breaks and boost cereal yields (Gladstones, 1970). Lupins thrive on low‐nutrient soils due to their ability to fix atmospheric nitrogen in symbiosis with beneficial bacteria and efficiently take up phosphorus from soils (Gladstones, 1970). Consequently, they are effective ecological pioneers and able to colonize extremely impoverished soils such as coastal sand dunes and new lava soils set down by recently erupted volcanoes (Lambers *et al*., 2013).

Lupins have emerged as both a human health food and food‐additive. Lupin seeds are rich in protein, ranging from 30% to 40% of whole seeds (Williams, 1979), with very little starch compared to other major grain legumes, for example chickpea and soya bean. The narrow‐leafed lupin kernel contains 40%–45% protein and 25%–30% dietary fibre, and low fat and carbohydrate content (Lee *et al*., 2006). An important property of lupin kernel flour is as a food‐additive (e.g. in bread); it increases satiety (thus reducing energy intake) and reduces insulin resistance, which are valuable properties in the context of the rising incidence of obesity and diabetes (Lee *et al*., 2006). Furthermore, lupin flour is increasingly used as a nongenetically modified alternative to soya bean products and is used to produce gluten‐free foods such as pasta. Studies on lupin seed proteins have provided valuable information on their number and RNA/protein expression patterns (Foley *et al*., 2011, 2015) as well as demonstrated that specific members are able to reduce glycaemia to comparable levels as achieved with metformin, a widely used hypoglycaemic drug (Magni *et al*., 2004).

Lupins belong to a single genus, *Lupinus*, in a legume clade known as the genistoids, which are believed to have diverged early in the evolution of papilionoid legumes (Lavin *et al*., 2005). There are an estimated 267 species of lupin distributed around the Mediterranean region (‘Old World’ lupins) and North and South America (‘New World’ lupins) (Drummond *et al*., 2012). Andean *Lupinus* species in particular show a rate of speciation unparalleled in the plant kingdom with broad morphological diversity ranging from small prostrate herbs to tall trees (Drummond *et al*., 2012; Hughes and Eastwood, 2006). Both annual and perennial species have found their niches in a vast array of ecological habitats across 100 degrees of latitude (Drummond *et al*., 2012). Together, these properties make this genus exceptionally useful for testing hypotheses relating to genome evolution, adaptation and speciation.

While wild lupin species were cultivated as far back as 2000 BC in the Mediterranean and Andean regions, domestication of lupin species was completed only in the 20th century (Gladstones, 1970). The most widely grown domesticated species today is narrow‐leafed lupin (*L. angustifolius*; NLL) (Lee *et al*., 2006). Its domestication was initiated in Germany in the early 20th century and completed in the 1960s in Australia with the development of the first fully domesticated cultivar with low alkaloid content, nonshattering pods, permeable seeds and early flowering. Since then, NLL cultivation has grown to span more than 600 000 hectares in over 20 countries (FAO, 2013).

Over the last decade, various legume genomes that utilized reference genetic maps to order and orient scaffolds for pseudomolecule assembly have been published, and these include those of *Medicago truncatula* (Young *et al*., 2011), chickpea (*Cicer arietinum*; Varshney *et al*., 2013), pigeon pea (*Cajanus cajan*; Varshney *et al*., 2012); common bean (*Phaseolus vulgaris*; Schmutz *et al*., 2014) and soya bean (G*lycine max*; Schmutz *et al*., 2010). In the latter two assemblies, synteny‐based refinement methods were used in addition to the dense genetic maps to order and orient assembled scaffolds into pseudomolecules (Schmutz *et al*., 2010, 2014). A range of genomic resources have also been produced in recent years for the study of lupins, particularly NLL. These include genetic maps for NLL and white lupin (Croxford *et al*., 2008; Kamphuis *et al*., 2015; Kroc *et al*., 2014; Nelson *et al*., 2010; Yang *et al*., 2013b) and large genomic insert libraries for NLL (Gao *et al*., 2011; Kasprzak *et al*., 2006). Transcriptomic resources have been developed for all four cultivated lupin species (Foley *et al*., 2015; Kamphuis *et al*., 2015; O'Rourke *et al*., 2013; Parra‐González *et al*., 2012; Secco *et al*., 2014; Wang *et al*., 2014). Preliminary draft genome data had been generated for NLL and were used to assist molecular marker design (Gao *et al*., 2011; Kamphuis *et al*., 2015; Yang *et al*., 2013a). In this study, we present the first high‐quality draft genome for a genistoid legume, narrow‐leafed lupin (2*n* = 40), report on a survey of its gene content and provide insights into its genome evolution, symbiotic relationships and host–pathogen interactions. Lupin, as a genus in the early‐diverging genistoid lineage in the papilionoid subfamily, serves as an outgroup for the many crop and model species in this subfamily—an outgroup that shares many characteristics of other papilionoid legumes (such as symbiotic nitrogen fixation), but with sufficient evolutionary distance to make inferences about the timing and histories of important molecular evolutionary events.

## Results and discussion

### The NLL genome assembly and gene features

The haploid genome size for NLL was previously estimated by flow cytometry to be 924 Mb (Kasprzak *et al*., 2006; Naganowska *et al*., 2003). K‐mer‐based estimation of genome size predicted a similar value of 951 Mb (Figure S1). Initial assembly of the Tanjil genome using only paired‐end Illumina data produced 191 701 scaffolds in 521 Mb, with an N50 of 10 137 and N50 length of 13.8 kb. The assembly was improved via scaffolding with additional paired‐end, mate‐pairs and BAC‐end data totalling an average coverage of 162.8 X (Table S1). This resulted in a contig assembly with 1 068 669 contigs, totalling 810 Mb or 85% of the K‐mer‐based estimated genome size. The final scaffold assembly after removing scaffolds less than 200 bp comprised 14 379 scaffolds totalling 609 Mb with a contig N50 length of 45 646 bp and scaffold N50 of 232 and scaffold N50 length of 703 Kb (Table 1).

**Table 1:** Summary of the narrow‐leafed lupin cv. Tanjil genome assembly. The assembly comprises scaffolds, the majority of which have been placed into pseudochromosomes based on genetic map and synteny data

Assembly statistics	Total length (bp)	Average length (bp)	Maximum length (bp)	Minimum length (bp)	N50	N50 length	Total # sequences	Total unknown N bases
Contigs	810 353 784	758	922 429	100	4 246	45 646	1 068 669	0
Scaffolds	609 123 749	42 362	4 089 732	200	232	703 185	14 379	4 078 848
Pseudochromosomes	470 424 067	23 521 203	36 457 581	16 251 777	8	24 697 652	20	3 351 285
Unplaced scaffolds	138 780 182	10 239	1 472 692	200	610	45 366	13 554	808 063

The NLL genome is highly repetitive (57% of the genome) (Table S2), with over half its repeats (32% of the genome) matching known transposable elements (TEs) (Table 2). Typical of most eukaryotes, TEs were most commonly long terminal repeats (LTRs) retrotransposons (28%), with DNA LTRs, long interspersed nuclear elements (LINE) and short interspersed nuclear elements (SINE) TEs comprising a relatively small proportion (4.8%, 2.7% and 0.1%, respectively). Noncoding RNA was estimated to comprise 0.1% of the genome (Table S3), the majority being ribosomal RNA (0.035%) and transfer RNA (0.012%), with predicted snRNA and miRNA representing 0.009% and 0.006%. Analysis of divergence between known TEs (Jurka *et al*., 2005) indicated a peak at ~30%, however the same analysis applied to *de novo* repeats produced a bimodal distribution with an additional less divergent peak at ~10% (Figure S2).

**Table 2:** Summary of transposon content in the narrow‐leafed lupin cv. Tanjil genome assembly

	Repbase TEs	TE Proteins	*De novo*	Combined TEs		% in Genome	Length (bp)	% in Genome
	Length (bp)	% in Genome	Length (bp)	% in Genome	Length (bp)			
DNA	8 983 926	1.47	7 351 979	1.20	23 429 353	3.83	29 084 889	4.76
LINE	8 299 104	1.35	10 841 081	1.77	13 051 653	2.13	16 438 300	2.69
LTR	79 075 250	12.95	90 533 453	14.83	154 738 027	25.35	172 348 763	28.23
SINE	66 384	0.01	0	0	483 328	0.08	544 025	0.09
Other	3917	0.000642	0	0	0	0	3917	0.000642
Unknown	0	0	2988	0.00049	134 545 040	22.04	134 548 028	22.04
Total	95 943 148	15.71	108 715 411	17.81	319 388 057	52.32	331 905 409	54.37

A total of 33 076 protein‐coding genes were annotated (Figure S3) after combining evidence from transcriptome alignments derived from five different tissue types (leaf, stem, root, flower and seed), protein homology, and *in silico* gene prediction (Table 3). Additionally, peptide data from proteomics analysis of leaf, seed, stem and root samples were mapped to both the translated gene annotations and the 6‐frame translation of the whole‐genome assembly (Bringans *et al*., 2009) (Table S4). Proteogenomic comparison of peptide‐mapping versus gene annotation supported between 94 and 1134 annotations per tissue type (Table S4), and provided valuable information on tissue localization for the products of these genes. InterPro terms were the most informative functional annotation assigned to NLL proteins with 26 580 (80.4%) proteins annotated (Table S5). Comparing gene counts for Interpro terms in NLL to other plant species (source: PLAZA 3.0 (Van Bel *et al*., 2011)) via Fisher's exact test, numerous Interpro terms were over‐represented in NLL and were often significantly higher than most species, excepting *G. max* (Data S1). However, in a few cases, NLL InterPro terms were more abundant versus all species including *G. max*. These included tyrosine protein kinases, photosystem II cytochrome b559, porins and microtubule‐associated proteins. The NLL assembly was also depleted in genes with InterPro terms corresponding to NBS–LRR proteins, DNA helicases, peptidase C48, hAT transposases and certain transcription factors.

**Table 3:** Summary of predicted protein‐coding gene annotations of narrow‐leafed lupin and their supporting evidence types

Gene set		Number	Average transcript length (bp)	Average CDS length (bp)	Average exon per gene	Average exon length (bp)	Average intron length (bp)
*De novo*	*AUGUSTUS*	34 525	2 983.98	1 252.69	5.49	228.06	385.36
	*GENSCAN*	29 436	10 570.55	1 367.30	6.22	219.52	1 760.19
	*A. thaliana*	48 717	2 815.40	968.47	3.72	260.12	678.23
Homolog	*C. cajan*	46 735	2 422.52	929.51	3.90	238.24	514.57
	*C. arietinum*	42 856	4 349.29	1 125.34	4.04	278.37	1 059.62
	*G. max*	39 433	3 648.01	1 245.43	4.55	273.19	675.09
	*M. truncatula*	61 321	2 454.66	843.16	3.10	271.33	764.67
	*P. vulgaris*	68 168	1 936.06	786.66	3.23	242.93	513.54
EST		1 795	2 134.32	606.22	3.16	191.27	704.40
*GLEAN*		32 413	3 568.05	1 305.97	5.58	233.78	493.22
RNA‐seq		49 946	2 309.00	803.54	4.01	199.94	498.02
Final set		33 076	3 673.44	1 289.14	5.52	233.52	488.41

### Chromosome‐level analysis of the NLL genome assembly

Enhanced genetic map data were used to place NLL scaffolds in a chromosomal context. Fluidigm assays yielded 469 transcriptome‐derived SNPs that were polymorphic in the RIL population (*n* = 153) derived from a domestic (83A:476) by wild (P27255) cross. An additional 8668 DArTSeq molecular markers, including 4767 presence/absence variants (DArT_PAV markers) and 3901 SNPs (DArT_SNP markers) were also applied. When combined with 830 previously reported sequence‐associated marker loci and seven trait loci, a total of 9972 loci (Data S2) were used to generate the improved map (Data S3), which comprised 20 linkage groups that correspond to the haploid chromosome complement of NLL (Lesniewska *et al*., 2011). The genetic map covers 2500.8 cM, with an average interval size of 0.85 cM between 2959 nonredundant framework loci (Table S6). This map incorporated for the first time a small orphan cluster of markers into linkage group 20 (Kamphuis *et al*., 2015) and has evenly distributed linkage group lengths (cM) (Figure S4A) and average interval sizes (cM) (Figure S4B).

A combination of 7707 markers physically mapped unambiguously to the scaffold assembly, including the following new markers: 3492 DArTSeq markers with presence–absence polymorphism, 2975 DArTSeq markers with SNP polymorphism, 555 Fluidigm markers with SNP polymorphism and 685 other previously reported PCR‐based markers (Gao *et al*., 2011; Kamphuis *et al*., 2015; Kroc *et al*., 2014) (Data S2). Twenty pseudomolecule sequences, ranging from 16.2 to 36.5 Mb, were built from 825 scaffolds. The pseudomolecule assemblies total 470 424 067 bp (77.2% of the full assembly length) (Table 1). Of these scaffolds, 820 were anchored to linkage groups and provisionally ordered and oriented using the high‐density marker resource, and five were added on the basis of synteny comparisons, using all‐by‐all dot plot comparisons between the NLL pseudomolecules and remaining unplaced scaffolds, and five other legume genomes (*Glycine max*,* Lotus japonicas*,* Medicago truncatula* and *Phaseolus vulgaris*; Data S6). The five added scaffolds comprised 2 004 769 bp or 0.4% of the pseudomolecule length. Additionally, when marker resolution was insufficient to confidently order and orient scaffolds (primarily in pericentromeric regions, where recombination rates are very low), synteny with the species above was considered in the scaffold order and orientation, under the assumption that discontinuities in genomic synteny that occur precisely at NLL scaffold boundaries are likely due to misorientation or local misplacement.

### Genome assembly validation and comparison to previous draft assembly

To validate the quality of the genome assembly a CEGMA analysis (Parra *et al*., 2007) was conducted to identify whether the majority of core eukaryotic genes are present in the assembly. This showed 235 complete and eight partial core eukaryotic genes were present in the assembly which equates to 98.0% or 243 genes of the gene set of 248 genes (for details and the missing protein KOG id's see Data S4). The transcriptome data for five different tissue types was aligned to the NLL assembly and for four of the five datasets 98.5%–99.0% of reads mapped back to the assembly (Table S7), suggesting the majority of the gene‐rich space of the NLL genome is captured in the assembly. For the root transcriptome 89.1% aligned back to the assembly, which could be due to contamination from the soil or soil microbes. Furthermore, of the 33 076 genes in the predicted gene set of the current assembly, 1.8% (596 genes) are absent in the previous draft assembly from 2013% and 47.5% (15 703 genes) had partial hits, whereas 50.7% (16 777 genes) had 100% complete alignment in the previous assembly. In conclusion, over 98% of the gene‐rich space is captured in our assembly and it is a significant improvement of the fragmented draft assembly from (Yang *et al*., 2013b) which had a scaffold N50 of 7319 scaffolds compared to 232 scaffolds for this assembly (Table 1).

CoReFinder (Collapse/Repeat Finder) was applied to 20 pseudochromosomes and unplaced scaffolds greater than 10 kb, and a total of 14 923 collapsed regions of 3 462 044 bp (0.58% of the genome) were identified (Data S5). In addition, a total of 66 301 repeated regions of 23 699 757 bp (3.89% of the genome) were identified. A copy number estimate of the repeated regions was also performed and ranged from 1.58 (pseudochromosome NLL‐01) to 171.60 (Scaffold_486) (Data S5). In conclusion, the assembly captures the majority of the gene space (~98%) and shows a low level of collapsed genes.

### Comparative genomics across legume species

**Figure 1:**
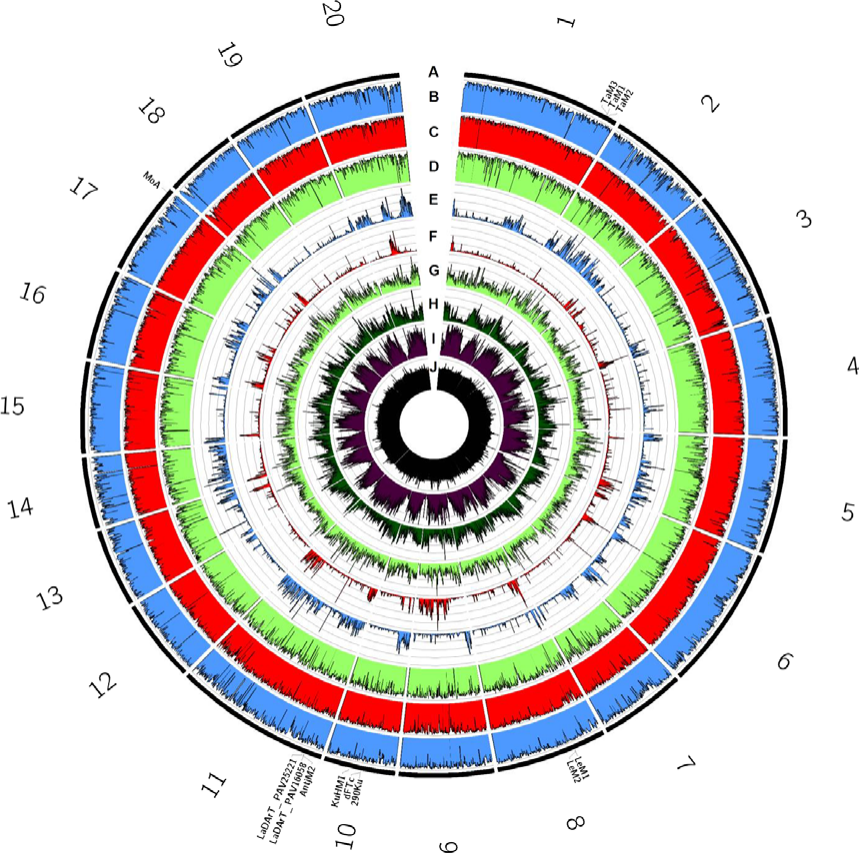
Summary of sequence variability in narrow‐leafed lupin lines Unicrop, 83A:476 and P27255, relative to pseudochromosomes (corresponding to linkage groups) of the reference genome of cv. Tanjil. (A) Pseudochromosomes (black), with sequence‐based genetic markers relevant to this study highlighted. (B–D) Per cent of 100‐Kb windows covered by ≥5× read depth for resequencing data from lines Unicrop (b, blue), 83A:476 (C, red) and P27255 (D, light green). (E–G) Density of polymorphic sequence sites ranging from 0 to 20 000 variants/Mb calculated within 100‐Kb windows, for lines Unicrop (E, blue), 83A:476 (F, red) and P27255 (G, light green). (H) Per cent of 100‐kb windows representing annotated genes in cv. Tanjil (dark green). (i) Per cent of 100‐kb windows represented annotated repetitive DNA in cv. Tanjil (purple). (j) Per cent G:C content ranging from 0% to 50%, calculated in 100‐Kb windows, in cv. Tanjil (black).

Resequencing of additional NLL lines at 51.5–59.2× coverage (Table S8) allowed comparisons of sequence variation across the NLL lines Unicrop (early domesticated cultivar), 83A:476 and P27255 (wild accession), relative to the pseudochromosomes of the reference cv. Tanjil (Figure 1). This indicated that the wild P27255 was significantly divergent across all regions of the genome with 216 167 indels and 3 053 917 SNPs (Table S8). In contrast, domesticated lines exhibited lower levels of diversity overall with 47 113 indels and 606 035 SNPs for line 83A:476 and 81 375 indels and 1 099 966 SNPs for cultivar Unicrop. Several trait‐associated markers (anthracnose and phomopsis resistance, flowering time, bitterness, pod shattering) could be mapped onto pseudochromosomes, facilitating ‘reverse‐genetic’ nomination of candidate genes for disease resistance and domestication traits (Table S9).

Comparison of orthologous gene content across multiple plant species highlighted a significant proportion of proteins that are conserved between NLL and four other legume species (Figure S5, Table S10). Among these species, lupin possesses a relatively high number of expanded paralogous genes (Figure S6), second only to *Glycine max*—likely due in both cases to independent whole‐genome duplication (WGD) and whole‐genome triplication in the *Glycine* and *Lupinus* lineages, respectively.

We find clear evidence of a whole‐genome triplication (WGT) in the genistoid lineage. This is inferred on the basis of synteny comparisons between NLL and itself and between NLL and other sequenced legume genomes (Data S6). Dot plots between NLL and another legume genome frequently show three strong, overlapping synteny blocks when these are viewed with respect to the other legume genome, or two blocks in the NLL self‐comparison (with the third copy visible as the NLL self‐match on the main diagonal). For the genomes *Lotus japonicus*,* Medicago truncatula* and *Phaseolus vulgaris*, the proportions of the NLL genome with a ‘synteny coverage depth’ of three with respect to the other genome are 21.4%, 21.0% and 13.2%, respectively (Table S11), while in comparisons going the ‘other way’ (with respect to NLL), the proportion of the genome with synteny coverage depth of three is negligible: 0.62%, 1.08%, 1.73%. In contrast, the proportion of those genomes with coverage depth at two (with respect to NLL) is high (14.2%, 27.5% and 28.6%), as expected, due to the papilionoid WGD (Table S11). In comparisons with *Glycine max*, the proportion of the genome with a ‘synteny coverage depth’ of three is 12.7% with respect to *Glycine*, while going the other way (with respect to NLL), the fourfold synteny coverage depth is greater than the threefold coverage depth (22.9% vs. 12.6%), as expected due to the additional WGD in the *Glycine* lineage.

Divergence times between *Lupinus* and other papilionoid legumes were calculated based on accumulation of synonymous changes between orthologous gene pairs between species (Figure S7), using a known species phylogeny and rooting the tree at the papilionoid WGD. The galegoid clade, containing *Lupinus*, is known to have originated near the base of the papilionoid subfamily (Lavin *et al*., 2005). If the papilionoid WGD immediately preceded the papilionoid radiation (Cannon *et al*., 2015), at ~58 Mya (Lavin *et al*., 2005), then we estimate the genistoid lineage separated from the other papilionoid legumes at ~54.6 Mya, and the whole‐genome triplication to have occurred in the genistoid lineage at ~24.6 Mya (Figure S7; Data S7).

These time estimates assume constant rates of synonymous nucleotide changes before and after the WGD. Additional taxon sampling in the Genisteae would be needed to refine the WGT timing; however, it is clear that the genistoid WGT is considerably older than the *Glycine* WGD, as Ks values for the WGT and WGD peaks are more than twofold greater in *Lupinus* than in *Glycine* (0.3 vs. 0.12).

**Figure 2:**
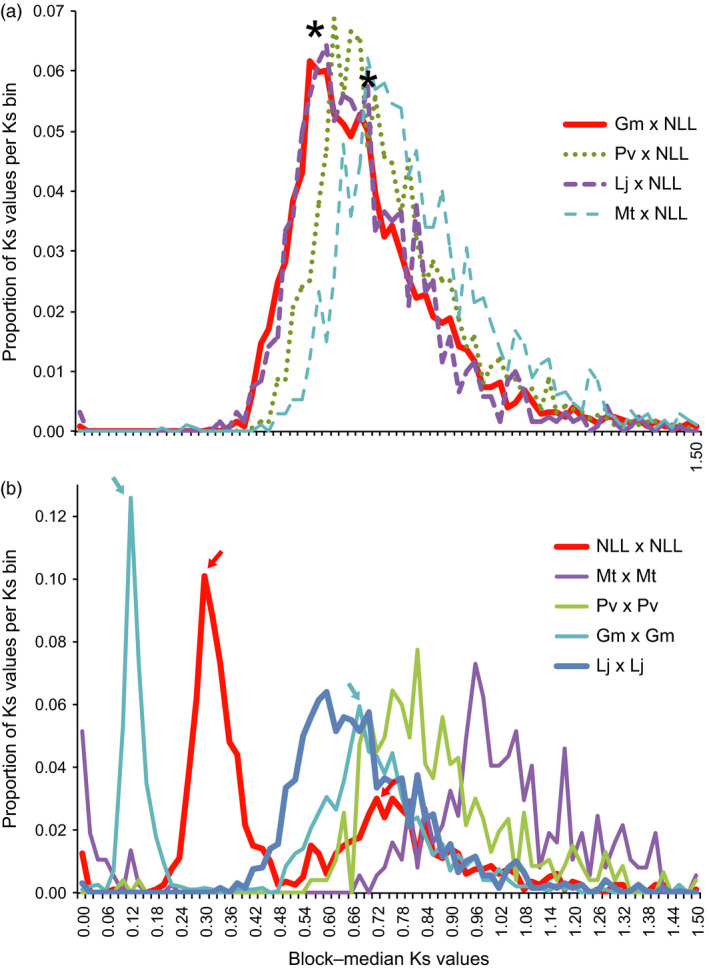
Synonymous substitution (Ks) analysis, showing proportion of values per Ks bin. Ks values are medians from synteny blocks for the indicated comparisons, and values in these plots are scaled to the total number of Ks counts for each comparison. (a) Orthologous comparisons between narrow‐leafed lupin (NLL) and *Glycine max* (Gm, red line), *Phaseolus vulgaris* (Pv, green dots), *Lotus japonicus* (Lj, purple dashed line) and *Medicago truncatula* (Mt, blue dashed lines). Asterisks show a primary peak for the speciation‐derived orthologs, and a probable smaller secondary peak for the papilionoid whole‐genome duplication‐derived ‘old orthologs’. (b) Paralogous genome self‐comparisons for narrow‐leafed lupin, *Medicago truncatula*,* Phaseolus vulgaris*,* Glycine max* and *Lotus japonicus*. Coloured arrows show two peaks in both *Glycine max* and narrow‐leafed lupin: the first peak in each case represents independent whole‐genome duplications in these lineages (*Glycine* at ~11 Mya and *Lupinus* at ~24 Mya), and the second peaks correspond to the shared papilionoid whole‐genome duplication. See Figure S7 and Data S7 for additional rate and date estimations.

From Ks analyses, we infer that the *Lupinus* lineage has accumulated point mutations at a rate similar to *Lotus* and *Glycine*, but more slowly than for *Phaseolus* or *Medicago*. This is apparent in papilionoid WGD peaks present at ~0.7 to 1.0, in self‐comparisons between paralogs (Figure 2). Furthermore, a WGT is evident in the genistoid lineage at around Ks ~0.3. This compares with the *Glycine* WGD peak at 0.12 and the papilionoid WGD at ~0.74 in *Lupinus* or ~0.68 in Glycine. If the papilionoid WGD occurred at ~58 Mya (Cannon *et al*., 2015; Lavin *et al*., 2005), then, assuming constant rates in this lineage, the genistoid WGT would have occurred at around 24.6 Mya.

Synteny comparisons with other sequenced legume genomes show extended regions of homology on all chromosomes, retained since divergence from the common ancestor of *Lupinus* and the other papilionoid species, which occurred ~55 Mya. For example, blocks spanning more than 6.4 million bases remain between soya bean and NLL (Table S12; Data S6, Data S7). Comparisons between NLL and soya bean generally show at least threefold synteny for NLL synteny viewed on soya bean as the reference, and at least fourfold synteny for soya bean synteny viewed on NLL as the reference, as both soya bean and NLL experienced the papilionoid WGD at ~58 Mya, and independent WGD at ~11 and WGT 24.6 Mya, respectively (see all‐by‐all chromosome dot plots for the NLL chromosomes compared to other NLL chromosomes and soya bean chromosomes in Data S6, and synteny depth coverage in Table S11). However, blocks are more degraded in NLL than soya bean. From the soya bean self‐comparison, in the recent and papilionoid WGDs, the longest remaining blocks are 12.8 million and 3.46 million bases, respectively, while from the NLL self‐comparison, in the recent and papilionoid WGDs, the blocks are 5.6 and 1.4 million bases, respectively. Average block lengths follow similar patterns, with the average ‘old’ (papilionoid) blocks from soya bean being 1.47 times longer than in NLL. The somewhat greater degradation in gene order in NLL is consistent with greater loss of paralogous genes (and decreased total gene count) in NLL than in soya bean.

### Relating NLL gene content to industry‐relevant phenotypes

Analysis of the annotated gene set using InterPro and Go‐terms (Data S8) coupled with the dense reference genetic map (Data S3) allowed the nomination of candidate genes for phenotypes segregating in the recombinant inbred line (RIL) population. A major disease pressure on lupins, including NLL, is anthracnose (caused by *Colletotrichum lupini*). The cultivar Tanjil is resistant to anthracnose, and a single dominant resistance gene (*Lanr1*) maps to linkage group 11 (Kamphuis *et al*., 2015; Yang *et al*., 2013b). Using our new genetic map (Data S3), we refined the location of *Lanr1* to a single scaffold (Scaffold_133), between flanking markers LaDArT_PAV20595 and LaDArT_PAV25221 (Table S9). This region spans 388 kb, harbours 5 cosegregating markers and contains 41 predicted genes (*Lup005013.1‐Lup005054.1*) including an NLR resistance gene (*Lup005042.1*). Alignment of *Lup005042.1* sequence from the four parents of the two RIL populations used to fine‐map the location of *Lanr1* showed complete conservation for resistant lines Tanjil and 83A:476, but considerable divergence to susceptible lines Unicrop and P27255 (Figure S8), thus making *Lup005042.1* a good candidate for *Lanr1*.

Legumes typically undergo important symbiotic relationships with other organisms. This includes associations with beneficial bacteria to form rhizobium–legume symbiosis (RLS) and with beneficial fungi to form arbuscular mycorrhizal symbiosis (AMS). Some of the genes required for a successful association are shared by both types of symbioses, and it is believed that the evolutionary younger RLS recruited part of the genetic programme of the more ancient AMS (Parniske, 2008). Around 80% of land plants can form AMS, but some lineages have lost this ability along with some of the genes required to establish this relationship (Delaux *et al*., 2014). Among legumes, lupins are unique, because they can form RLS but are unable to form AMS. This has been used to identify genes that are shared between both symbiotic associations (Bravo *et al*., 2016; Delaux *et al*., 2014). The NLL genome was screened for the presence of AMS genes and was found to include 20 of 38 characterized mycorrhizal association genes. These included genes involved in rhizobium–legume symbiosis, or the biosynthesis, regulation or transport of plant hormones. However, NLL lacked key genes required specifically for AM symbiosis (in *italics* in Table 4) but not nodulation, including *SbtM1*,* SbtM3*,* HA1*,* EXO70I, RAM2*,* PT4*,* STR1*,* STR2*,* RAM1*,* ERF1*,* RAD1, DIP1, FatM, KIN2, KIN3, KIN5, RFCb* and *CYT733A1* (Table 4; Data S9). The only exception was PP2AB'1, which so far is known only to be required for AMS (Charpentier *et al*., 2014), but may play other, yet to be discovered roles in lupin biology. During nodulation, lupins become infected by rhizobia via intercellular penetration rather than through intracellular infection threads, as do most other legumes (González‐Sama *et al*., 2004). Short‐infection‐thread‐like structures have been observed in cortical cells, but their importance is not clear (González‐Sama *et al*., 2004; James *et al*., 1997; Tang *et al*., 1992). Despite this, all genes known to be required for rhizobial infection were present in NLL (Table 4), suggesting fundamentally conserved mechanisms underlying different infection modes.

**Table 4:** Overview of genes associated with arbuscular mycorrhizal and rhizobial associations in the genomes of *Medicago truncatula* and narrow‐leafed lupin

Symbiotic component	Gene product	Medicago	Lupin	Reference^a^
NUP85	Nucleoporin	*Medtr1g006690*	*Lup020970.1*	1
NUP133	Nucleoporin	*Medtr5g097260*	*Lup029707.1*	2
NENA	Nucleoporin	*Medtr6g072020*	*Lup022917.1*	3
MCA8	Calcium pump	*Medtr7g100110*	*Lup028615.1 Lup006231.1 Lup028310.1 Lup018698.1*	4
DELLA1	Transcriptional regulators	*Medtr3g065980*	*Lup023873.1 Lup029445.1*	5
DELLA2	Transcriptional regulators	*TC182493*	*Lup007545.1 Lup009138.1*	5
CCD7	Carotenoid cleavage dioxygenase	*Medtr7g045370*	*Lup003751.1*	6
CCD8	Carotenoid cleavage dioxygenase	*Medtr3g109610 Medtr7g063800*	*Lup028507.1*	6
PDR1	ABC transporter	*Medtr3g107870 Medtr1g011640 Medtr1g011650*	*Lup013990.1 Lup001244.1*	7
D27	Carotenoid isomerase	*Medtr1g471050 Medtr7g095920*	*Lup011456.1 Lup018644.1*	8
SUT2	Sucrose transporter	*Medtr8g468330*	*Lup016593.1*	9
DMI1 (Pollux)	Cation channel	*Medtr2g005870*	*Lup014919.1*	10
NSP1	GRAS transcription factor	*Medtr8g020840*	*Lup007304.1*	11
NSP2	GRAS transcription factor	*Medtr3g072710*	*Lup012083.1*	12
DMI3 (CCaMK)	Calcium/calmodulin‐dependent protein kinase	*Medtr8g043970*	*Lup001774.1*	13
DMI2 (SYMRK/NORK)	Receptor‐like kinase	*Medtr5g030920*	*Lup025527.1*	14
Castor	Cation channel	*Medtr7g117580*	*Lup029273.1*	15
VAPYRIN	MSP and ANK repeat‐containing protein	*Medtr6g027840*	*Lup000011.1 Lup001531.1*	16
IPD3 (Cyclops)	Coiled‐coil domain containing protein	*Medtr5g026850*	*Lup027672.1*	17
NFP	LysM receptor‐like kinase	*Medtr5g019040*	*Lup012981.1*	18
PP2AB'1	Protein phosphatase 2A	*Medtr1g112940*	*Lup024672.1*	19
LYK3	LysM receptor kinase	*Medtr5g086130*	*Lup018960.1*	20
ERN1	Transcription factor	*Medtr7g085810*	*Lup000007.1*	21
ERN2	Transcription factor	*Medtr6g029180*	*Lup009942.1*	22
NIN	Transcription factor	*Medtr5g099060*	*Lup029716.1*	23
NF‐YA1	Transcription factor	*Medtr1g056530*	*Lup000323.1*	24
NF‐YA2	Transcription factor	*Medtr7g106450*	*Lup019646.1*	25
RPG	Coiled‐coil protein	*Medtr1g090807*	*Lup001677.1*	26
LIN	E3 ubiquitin ligase	*Medtr1g090320*	*Lup001700.1*	27
PUB1	E3 ubiquitin ligase	*Medtr5g083030*	*Lup029507.1*	28
SUNN	LRR receptor kinase	*Medtr4g070970*	*Lup003404.1*	29
NPL	Pectate lyase	*Medtr3g086320*	*Lup011017.2*	30
CRE1	Cytokinin receptor	*Medtr8g106150*	*Lup008799.1*	31
FLOT4	Flotillin	*Medtr3g106430*	*Lup030707.1*	32
SYP132A	Syntaxin	*Medtr2g088700*	*Lup029417.1 Lup030298.1*	33
SbtM1	*Subtilisin‐like protease*	*Medtr5g011320*		34
SbtM3	*Subtilisin‐like protease*	*Medtr5g011340*		34
HA1	*ATPase*	*Medtr8g006790*		35,36
Exo70	*Exocyst complex protein*	*Medtr1g017910*		37
RAM2	*GPAT*	*Medtr1g040500*		38
PT4	*Phosphate transporter*	*Medtr1g028600*		39
STR1	*Half‐ABC transporter*	*Medtr8g107450*		40
STR2	*Half‐ABC transporter*	*Medtr5g030910*		40
RAM1	*GRAS transcription factor*	*Medtr7g027190*		41
ERF1	*Transcription factor*	*Medtr7g009410*		42
RAD1	*GRAS transcription factor*	*Medtr4g104020*		43
DIP1	*GRAS transcription factor*	*Medtr8g093070*		44
FatM	*Acyl‐(ACP) thioesterase*	*Medtr1g109110*		45
KIN2	*Protein kinase*	*Medtr4g129010*		45
KIN3	*Protein kinase*	*Medtr7g116650*		45
KIN5	*Serine–threonine protein kinase*	*Medtr3g104900*		45
RFCb	*Replication factor C*	*Medtr3g118160*		45
CYT733A1	*P450 enzyme*	*Medtr6g034940*		45

a References for each of the functionally characterized genes in relation to either AM symbiosis or Rhizobia symbiosis can be found in Data S10.

We also examined genes involved in flowering time as early flowering is an important trait in NLL (Berger *et al*., 2013). Most genes and gene families prominent in flowering time control and light signalling in other dicot species were represented in NLL (Table S13), with notable exceptions. These included the FLC clade of vernalization‐responsive MADS‐domain proteins, which appears to be broadly absent from legume genomes including NLL (Hecht *et al*., 2005). Other genes appeared absent from NLL despite their presence in other papilionoid legumes (Table S13). These included the red light photoreceptor gene *PHYE*, which is present in Medicago and soya bean but absent in pea (Hecht *et al*., 2005; Platten *et al*., 2005; Schmid *et al*., 2003; Yant *et al*., 2010), suggesting it may have been lost more than once during legume evolution. A more striking case is the *FT* family which appears to consist of three relatively ancient clades in papilionoid legumes, *FTa*,* FTb* and *FTc*, with the *FTa* clade further divided into *FTa1* and *FTa3* subclades (Hecht *et al*., 2011). Genes in the *FTa1* and *FTb* clades have significant roles in flowering time control in other legumes (Hecht *et al*., 2011; Kong *et al*., 2010; Laurie *et al*., 2011; Zhai *et al*., 2014), but both groups of genes are absent from the NLL draft assembly, which has only duplicated copies of *FTa3* and *FTc* genes. Furthermore, microsynteny analysis between chickpea, common bean, medicago, soya bean and NLL showed conservation of the genes flanking the *Ft* genes in legumes with the *FTa1*,* FTa2* and *FTb* absent in the NLL genome assembly, whereas *FTc1* and *FTa3* are present in the Tanjil assembly (Figure S9). The *FTa1* and *FTb* clade genes are also not found in any of the comprehensive NLL transcriptome datasets (Kamphuis *et al*., 2015). This implies that the strong vernalization response of NLL (Berger *et al*., 2012) involves a mechanism distinct from that in *Medicago truncatula* where *FTa1* is the major target (Laurie *et al*., 2011).

## Conclusion

The comprehensive draft assembly of NLL (cultivar Tanjil) is the first representative of the genistoid clade of *Papilionoideae* legumes and will support further whole‐genome analysis of other species in this important clade. Resequencing of additional lupin lines and in‐depth transcriptome sequencing revealed widespread polymorphisms that were used to generate a dense reference genetic map. These resources are accessible through the Lupin Genome Portal (http://www.lupinexpress.org) which includes interactive BLAST, GBrowse and CMap interfaces (Donlin, 2009; Priyam *et al*., 2015; Youens‐Clark *et al*., 2009) and provides a platform for genome‐wide association studies and genomics‐based breeding approaches. The knowledge of germplasm diversity, and capacity for reverse genetics facilitated by the dense genetic map and pseudomolecule assembly can accelerate future breeding of elite cultivars. This will fortify efforts to improve lupins as human health food crops and increase yield stability and productivity of lupins for farmers worldwide.

## Experimental procedures

### Library preparation and sequencing

Paired‐end Illumina gDNA libraries of 100 bp length and 170, 500 and 800 bp insert sizes were generated (82.2× coverage). This was complemented by mate‐paired libraries of 50 bp read length and 2, 4, 10, 20 and 40 Kb insert sizes generating a total of 150.41 Gb or 162.8× coverage. Illumina sequence reads were trimmed for adapter and low‐quality sequences via CutAdapt v1.1 (min length 25 bp, rounds 3, match length 5 bp) (Martin, 2011). Mate‐paired libraries were filtered for contaminating paired‐end reads by merging pairs of reads with overlapping 3′ sequences via FLASH v1.2.2 (Magoč and Salzberg, 2011). Additional RNA‐seq Illumina data used in this project to complement genome sequence data were described in a previous study (Kamphuis *et al*., 2015). The total genome size for narrow‐leafed lupin was estimated by performing a 17‐mer frequency analysis of genomic paired‐end libraries via Kmerfreq (Liu *et al*., 2013), using the following equation: total genome size = (K‐mer frequency/primary peak depth).

### Genetic mapping

To assign scaffolds to linkage groups, we developed additional transcriptome‐derived SNP markers compatible with the Fluidigm microfluidic array platform as previously described (Kamphuis *et al*., 2015) to add to the 1475 loci of the previous reference genetic map. These new Fluidigm SNP assays (469) were used to genotype the same 153 recombinant inbred lines (RILs) developed from a cross between 83A:476 (an Australian breeding line) and P27255 (a wild accession from Morocco) used previously to generate a genetic map (Nelson *et al*., 2006). Additionally, DArTSeq analysis (Diversity Arrays Technology, Canberra) was performed, resulting in a further 3901 SNP‐polymorphic markers and 4765 markers polymorphic by allele presence/absence. These new markers, together with the 830 previously reported STS markers, giving a total of 9972 markers and seven phenotypic trait loci (Kamphuis *et al*., 2015), generated an improved genetic map prepared with the aid of MultiPoint 3.1 (MultiQTL Ltd., Haifa, Israel) using the approach detailed in our previous study (Kamphuis *et al*., 2015).

### *De novo* genome assembly and validation

Paired‐end Illumina data were assembled via SOAPdenovo2 (Luo *et al*., 2012) producing an initial assembly that was further scaffolded by SSPACE2 v2.0 (Boetzer *et al*., 2011), progressing iteratively through paired‐end (170, 500 and 800 bp) and mate‐paired (2, 4, 10, 20 and 40 Kb) sequence libraries in order of increasing insert size. Five rounds of scaffolding were performed for each insert library, followed by five rounds of gap‐closing via BGI GapCloser (Luo *et al*., 2012) using paired‐end sequences only. Further scaffolding was performed using BAC‐end sequence data (insert size ~100 Kb) (Gao *et al*., 2011), via Bambus (range 50–400 000 insert) (Pop *et al*., 2004). The length of assembly ‘gaps’ (i.e. unknown stretches represented by runs of >10 ‘N’ bases) was corrected to a uniform 100 bp. Scaffolds were screened for simple repeats via RepeatMasker (‐no_is ‐norna ‐noint) (Smit *et al*., 1996–2010) and tandem repeats finder (2 7 7 80 10 50 50 ‐f ‐d ‐m ‐h) (Benson, 1999). Sequences <200 bp length or with >=50% repetitive, simple repeat or unknown N bases were removed from the assembly as per GenBank requirements. The assembly was then validated versus the new genetic map generated in this study. Sequence‐based genetic markers were mapped to scaffolds via ePCR (Schuler, 1997) and BLASTN ((Altschul *et al*., 1990); e ≤ 1‐e05). Marker location on scaffolds was determined preferentially by ePCR (max. 2 gaps, 2 mismatches, amplicon range 10–1000 bp for markers designed for the Fluidigm platform, 10–5000 bp for other markers), where *in silico* PCR produced a single amplicon for the match with the minimum possible hamming distance (mismatches+gaps). Where *in silico* PCR could not determine an unambiguous marker location, the locations of unambiguous top BLASTN hits for known marker amplicon sequences to the scaffolds were used instead. Scaffold joins were compared to marker order on the genetic map, and where a conflict was found, preliminary scaffolds were split on all ‘gaps’ located between conflicted markers. Whole‐genome alignments to the *Glycine max* genome assembly using promer and mummerplot (Kurtz *et al*., 2004), generally filtering at 90% identity and requiring maximum unique matches (–mum), were also used to manually split scaffolds where ‘macrosynteny’ was observed and this did not conflict with genetic map data. This final filtered and validated set of scaffolds was then assembled into pseudochromosomes based on the 20 linkage groups of the genetic map. Where possible, scaffolds were assigned to linkage groups in the order of their constituent markers on the map, reverse complemented if indicated by two or more markers. Synteny versus *G. max* was also used to manually place scaffolds on the map, particularly where abrupt disruption of synteny corresponded to neighbouring scaffold termini. Scaffolds placed on linkage groups were subsequently joined by uniform unknown gap lengths of 100 bp to form pseudochromosomes.

### Annotation of genes and other genome features

Transcriptome sequences for cv Tanjil were previously assembled by Kamphuis *et al*. (2015), and in this study additional transcriptomes for cv Unicrop and P27255 were generated by the same method. Annotation of gene structure in the cv Tanjil reference genome was predicted *de novo* using AUGUSTUS (Stanke *et al*., 2006) and GENSCAN (Burge and Karlin, 1997). Further support for gene annotations was provided through alignment to the genome assembly of EST sequences derived from GenBank EST records listed under the taxon ‘Fabaceae’ (Benson *et al*., 2013), and homology to proteins of *Arabidopsis thaliana* (Initiative, 2000), *Cajanus cajan* (Varshney *et al*., 2012), *Cicer arietinum* (Varshney *et al*., 2013), *Glycine max* (Schmutz *et al*., 2010), *Medicago truncatula* (Young *et al*., 2011) and *Phaseolus vulgaris* (Schmutz *et al*., 2014). *De novo* predictions were combined and curated with supporting homology and EST evidence via GLEAN (Elsik *et al*., 2007). RNA‐seq data were aligned to the genome via TopHat (Trapnell *et al*., 2009), assembled transcripts via Cufflinks (Trapnell *et al*., 2010) and predicted open reading frames according to transcript alignments. GLEAN results were aggregated with RNA‐seq‐supported gene models to produce the final gene set. Functional annotations were assigned to genes based on searches against Interpro (Quevillon *et al*., 2005), KEGG (Kanehisa and Goto, 2000), GO (Ashburner *et al*., 2000) and UniProt (The UniProt Consortium, 2013).

Repetitive DNA regions were predicted in the genome for both transposable elements (TEs) and tandem repeats. Annotation of TEs was based on homology and *de novo* methods. The homology approach used RepeatMasker v3.30 (Smit *et al*., 1996–2010) (with RepeatProteinMasker) to identify repeats matching known repeat sequences in Repbase v16.10 (Jurka *et al*., 2005). The *de novo* method predicted repetitive DNA via Repeatmodeler v1.0.5 (Smit and Hubley, 2010). Tandem repeats were predicted using Tandem Repeats Finder v4.04 (Benson, 1999).

### Proteogenomics

Samples of NLL were obtained from leaf, seed, stem and root tissues and protein extracted and subjected to iTRAQ by Proteomics International using to the iTRAQ protocol (Sciex, USA).

Spectral data were analysed using ProteinPilot^™^ 4.0 Software (Sciex) against query and decoy databases generated from both translated gene annotation and six‐frame‐translated open reading frames. The database of potential open reading frames was generated by obtaining the six‐frame translation of scaffolds via EMBOSS getorf (between stop codons, ≥10 aa in length). The spectral data were exported as XML files with proteogenomic mapping of peptides to scaffold and pseudochromosome sequences performed with CDSmapper (http://sourceforge.net/projects/cdsmapper/).

### Comparative genomics

#### Analysis of variation across the cv Tanjil genome

SNP and indel sequence variation was assessed across a panel of cultivars relative to the cv. Tanjil reference genome. NGS reads were aligned to the cv Tanjil reference genome via bowtie v 2.0.5 (–very‐sensitive) (Langmead and Salzberg, 2012), and variants were called via the Genome Analysis Tookit 3.4‐46 (McKenna *et al*., 2010). GATK was used to perform read deduplication via Markduplicates, then variant calling with HaplotypeCaller (–stand_call_conf 20 –stand_emit_conf 20 –min_pruning 5), producing variant data in VCF format (Danecek *et al*., 2011). Genome comparisons were visualized using Circos v0.67‐1 (Krzywinski *et al*., 2009).

Orthologous gene clusters were predicted via OrthoMCL (Li *et al*., 2003) comparing translated annotations of NLL to protein datasets from *C. cajan* (Varshney *et al*., 2012), *C. arietinum* (Varshney *et al*., 2013), *G. max* (Schmutz *et al*., 2010), *M. truncatula* (Young *et al*., 2011), *P. vulgaris* (Schmutz *et al*., 2014) and *A. thaliana* (Initiative, 2000).

Analysis of rates of silent‐site substitutions was carried out by searching all peptides against all others for the species *Lupinus angustifolius, Glycine max* (v 2.0)*, Lotus japonicus* (v 3.0)*, Medicago truncatula* (v 4.0) *and Phaseolus vulgaris* (v 1.0). Top respective matches were retained between each species per chromosome pairing (allowing for multiple total hits between two species for a given query gene), and within each species (for analysis of whole‐genome duplications). Then in‐frame alignments of coding sequences were made for each retained peptide alignment. From alignments of coding sequences, values for Ks, Ka and Ka/Ks were calculated using the ‘codeml’ method from the PAML package (Yang, 2007). Also from protein alignments, synteny blocks were inferred using DAGchainer (Haas *et al*., 2004). From the per‐gene‐pair alignments and the synteny blocks, median Ks values for blocks were calculated and used for Ks histogram peaks (Figure 2).

Ages of species divergences and whole‐genome duplications (Figure S7) were calculated from modal Ks peaks (Data S7), by treating initially unknown branch lengths in the known species/duplication tree as variables in a set of equations. The species/duplication tree was rooted at the papilionoid whole‐genome duplication, which predated the main papilionoid radiation (Cannon *et al*., 2015). A time of 58 Mya for the initial papilionoid radiation was assumed (Lavin *et al*., 2005). There were 11 unknown branch lengths in the tree in Figure S7, and sufficient data from the modal distances between and within species comparisons to solve for these unknowns algebraically.

To evaluate evidence for a whole‐genome triplication (WGT), synteny blocks were identified using DAGchainer, and synteny coverage depth was calculated using the BEDTools v2.25.0 (Quinlan and Hall, 2010) ‘coverage’ function to make comparisons between other genomes and NLL as the reference, or between NLL and each other genome as the reference. Coverage of synteny blocks was calculated at each nucleotide position using the ‐d option and summarized per coverage depth level.

For visual dot plot assessments of NLL compared with itself and with other legume genomes, we used promer and mummerplot from the MUMmer package (Kurtz *et al*., 2004), (v3.23) to make comparisons of translated nucleotide sequence, on genomic sequence that was masked for all except exonic sequence. The promer results were filtered to require at least 80% identity.

### Genome assembly validation and comparison to previous draft assembly

The quality of the Tanjil draft assembly was evaluated using the default parameters of CEGMA (Core Eukaryotic Genes Mapping Approach) v 2.5 (Parra *et al*., 2007).

CoReFinder (Collapse/Repeat Finder) is a differential comparative read mapping pipeline, which identifies and discriminates between collapsed and repeated regions in genome assemblies. Paired‐end reads with insert sizes of 170 bp, 500 bp and 800 bp totalling a coverage of 138.63× were aligned to the assembly using SOAPaligner v2.21 (Li *et al*., 2009b), with mapped reads reported in three ways via the ‘−r’ parameter: −r0 (reads that map uniquely), −r1 (reads that map to more than one location, but only one random hit is reported) and −r2 (report all hits) and converted to sorted .bam files using SAMtools v1.2 (Li *et al*., 2009a). The .bam files were then split into pseudochromosomes/scaffolds using BamTools v2.4.0 (Barnett *et al*., 2011), such that for each pseudochromosome/scaffold there were three .bam files corresponding to each mapping. The per‐base coverage was calculated for each .bam file using BEDTools v2.25.0 (Quinlan and Hall, 2010). The BEDTools output was merged such that each pseudochromosome/scaffold had a single tab‐delimited output file consisting of the name of the pseudochromosome/scaffold, the position, the per‐base coverage for −r0, the per‐base coverage for −r1 and the per‐base coverage for −r2.

For each pseudochromosome/scaffold, a custom R script was used on the tab‐delimited file to mine for collapsed and repeated regions by iterating through each position in the file. Any region where the median per‐base coverage of −r0, −r1 and −r2 was greater than twice the overall median coverage was flagged as ‘coll’ (collapsed). Any region where the coverage for −r0 was between 0 and 2, the coverage for −r1 was greater or equal to 2, and the coverage for −r2 was 0.5 times the overall median was marked as ‘rnc’ (repeated, non‐collapsed). Regions that were marked as ‘coll’ or ‘rnc’ that were within 100 bp of each other were merged using BEDTools.

NGS reads were aligned to the previous draft assembly and the current Tanjil assembly via bowtie v 2.0.5 (–very‐sensitive) (Langmead and Salzberg, 2012), and RNASeq datasets for the five tissue types were aligned to the assembly using TopHat v 2.0.9 (–b2‐very‐fast −r 50 –mate‐std‐dev 200 ‐i 20 ‐I 4000‐g 20 ‐report‐secondary‐alignments ‐m 0–min‐coverage‐intron 20 –coverage‐search –microexon‐search) to determine and compare the coverage of the various paired‐end, mate‐pair and RNASeq datasets in the two Tanjil draft assemblies.

### Comparison of gene function across Lupinus and other plant taxa

To observe general variation in gene function, functional annotations were assigned to the proteins of NLL cv Tanjil via Interproscan (Quevillon *et al*., 2005) and compared to those assigned in other plant species available from the PLAZA Dicots v3.0 and Monocots v3.0 databases (Van Bel *et al*., 2011). Fisher's exact test was applied to the number of genes assigned an Interpro term in NLL versus *Glycine max*, or the average of various groups of species: legumes, dicots, monocots and all available Viridiplantae (Data S1). In the Supplementary Data File provided, further filtering has been applied requiring an expansion in NLL (gene count fold change > 1) and a *P*‐value of ≤ 0.05.

To focus on variation in gene content relevant to arbuscular mycorrhiza and rhizobia association, a protein database was constructed which included the predicted proteins of NLL cv. Tanjil from this study, and 50 other land plant species (Bravo *et al*., 2016). This database was queried with proteins known to be involved in arbuscular mycorrhizal symbiosis in *Medicago truncatula* via BLASTP (Altschul *et al*., 1990), and the top 200 matches were used to create phylogenies. The protein models were aligned using MAFFT v7.205 (Katoh *et al*., 2002) with default values, and columns of the alignment that contained more than 50% gaps were eliminated. A phylogenetic tree was generated with FastTree v2.1.5 (Price *et al*., 2010) using the wag model of amino acid evolution. The presence or absence of NLL true orthologs was assessed through visual analysis of the topology of the phylogenies generated.

### Accession code

Genome sequence assembly and annotation data can be found in GenBank under BioProject ID: PRJNA299755 and is also available for download and interrogation via BLAST and GBrowse from the Lupin Genome Portal (http://www.lupinexpress.org).

## Author Contributions

JKH, MY, LGK, GG, MNN, SB, RF, L‐LG, SL, AM, JJ and KBS contributed to generation of genome sequence, transcriptome sequences, BAC‐end sequences, genetic mapping and physical mapping data and development of the genome browser; JKH, MY, LGK, GG, SC and JJ worked on genome assembly; JKH, MY and JJ contributed to the genome annotation; JKH, MY, LGK, MNN, AB, SB, RF, L‐LG, MJH, SL, C‐WL, AM, JM, JW and JJ contributed to the gene function analysis; JKH, MY, LGK, MNN, SC, GG, WH, PEB, BH, DE and JJ worked on genome analysis and comparative genomics; JKH, LGK and KBS wrote the manuscript with input from MY, MNN, CAA, SB, SC, MJH, AM, GM, JM, JM, DE and JJ; KBS; CAA, GM and KBS conceived and directed the project.

## Supporting information


**Figure S1** K‐mer‐based estimation of genome size based on frequency analysis of 17‐mers in paired‐end libraries. A primary coverage peak in 17‐mer frequency was observed at 39× coverage, which corresponded to a frequency of 37 098 706 666 and 44 885 658 200 bases. Using the equation (K‐mer frequency/Peak depth) the K‐mer frequency analysis estimated a total genome size of 951 248 889 bp or 951.2 Mb. This estimate is consistent with C‐value based estimates of 924 Mb.
**Figure S2** Summary of sequence divergence (100% – sequence identity) across DNA transposon and retrotransposon families (LTR, LINE and SINE) predicted in the narrow‐leafed lupin cv. Tanjil genome assembly relative to: (a) a representative repeat sequence in Repbase; or (b) *de novo* repeat family consensus sequence.
**Figure S3** Length distributions for gene annotation sub‐feature including mRNA (a), coding sequence (CDS) (b), exons (c) and introns (d).
**Figure S4** (a) Length (cM) of the 20 linkage groups of the 83A:476 × P27255 RIL genetic map using 2959 non‐redundant molecular markers*. (b) Average interval size (cM) of the 83A:476 × P27255 RIL genetic map using 2959 non‐redundant molecular markers*. *Out of the 9972 markers genotyped in the RIL population (*n* = 153) 2959 had unique (non‐redundant) centiMorgan positions in the genetic map. Of the 9972 markers 7707 physically mapped unambiguously to unique locations in the pseudomolecule assembly and were used to orientate and assign scaffolds to pseudomolecules.
**Figure S5** Venn diagram showing shared orthologous groups in narrow‐leafed lupin and other sequenced plant genomes.
**Figure S6** Protein orthology comparison of narrow‐leafed lupin (*L. angustifolius*) and other sequenced plant species.
**Figure S7** Estimation of divergence time for narrow‐leafed lupin (*L. angustifolius*) and selected other sequenced legume species. Red numbers are estimated divergence times from the present day in millions of years (Mya), derived relative to an assumed time of 58 Mya for the origin of the papilionoid clade and papilionoid whole‐genome duplication at effectively the same time. Black numbers on the branches are rates of synonymous‐site changes (Ks), calculated from modal Ks values between all paralogous genes between the species included above (Data S6). Asterisks mark whole‐genome duplications/triplication.
**Figure S8** Alignment of translated amino‐acid sequences corresponding to narrow‐leafed lupin cv. Tanjil locus *Lup005042.1* for anthracnose‐resistant (*Colletotrichum lupini*) NLL lines 83A:476 and Tanjil and susceptible NLL lines Unicrop and P27255. The translated amino‐acid sequences for 83A:476, P27255 and Unicrop were derived from the re‐sequencing data for these accessions/cultivars.
**Figure S9** Schematic representation of microsynteny analysis of the *FTa*,* FTb*, and *FTc* gene clusters in barrel medic (*M. truncatula*), chickpea (*C. arietinum*), common bean (*Phaseolus vulgaris*), soybean (*G. max*) and narrow‐leafed lupin (*L. angustifolius*) showing that *FTa1, FTa2* and *FTb* genes are absent in these microsyntenic regions of NLL. (a) *FTb* region microsynteny (b) *FTa1/2* and *FTc1/2* region microsynteny; (c) *FTa3/4* region microsynteny.
**Table S1** Summary of the total amount of sequence data generated for the *L, angustifolius* cv. Tanjil genome assembly and the average coverage per paired‐end and mate‐pair library, assuming an estimated genome size of 924 Mb based on C value prediction.
**Table S2** Summary of repetitive DNA regions predicted within the narrow‐leafed lupin genome.
**Table S3** Summary of non‐coding RNA genes predicted within the narrow‐leafed lupin cv. Tanjil genome assembly.
**Table S4** Summary of proteomics analyses applied to four tissues of narrow‐leafed lupin and the number of peptides and proteins identified at a 99% confidence interval.
**Table S5** Summary of functional annotations assigned to gene annotations in narrow‐leafed lupin cv. Tanjil.
**Table S6** Summary statistics for 20 narrow‐leafed lupin linkage groups NLL‐01 to NLL‐20 comprising 9965 molecular markers and 7 trait loci.
**Table S7** Read alignment of narrow‐leafed lupin cultivar Tanjil RNASeq data for five different tissue types, paired‐end and mate‐pair data to the genome.
**Table S8** Overview of the coverage and variants (insertions/deletions and SNPs) identified for the three re‐sequenced narrow‐leafed lupin lines.
**Table S9** Location of domestication traits and disease resistance genes in the genome assembly of narrow‐leafed lupin cv. Tanjil.
**Table S10** Summary of orthologous gene families in narrow‐leafed lupin and other sequenced plant species.
**Table S11** Synteny coverage depth for NLL vs. other genomes and for other genomes vs. NLL. Each row begins with a coverage depth (0, 1, 2, etc.). Percentages for a given coverage depth and a species indicate the proportion of the genome with the indicated synteny coverage depth, with respect to the indicated reference genome. For example, in table 11A, 21.4% of the Lotus genome is covered by three synteny features with NLL.
**Table S12** Values under “WGD Ks peaks” are the Ks bin values for the mode in Ks plots from the indicated species pairs, corresponding to the papilionoid whole‐genome duplication (WGD) in that Ks plot. Plots can be seen in Figure 2 and in Supplemental Data File 5. For example, for *Glycine*‐*Glycine*, this would be the second modal peak in the orthologous plot, Figure 2B (the most recent peak being the one from the WGD within the *Glycine* lineage). For *Glycine*‐*Lupinus*, the WGD peak is older than the speciation peak. This can be seen in Figure 2A, second asterisk. Values under “Speciation Ks peaks” are also inferred from Figure 2 and in Supplemental Data File 6. For example, for *Glycine*‐*Lupinus*, the speciation peak can be seen in Figure 2A, first asterisk. Values under “Recent independent WGD peaks” are seen in Figure 2B for *Glycine*‐*Glycine* and *Lupinus*‐*Lupinus*.
**Table S13** Summary of genes and gene families prominent in flowering time control and light signalling in other dicot species present or absent in the narrow‐leafed lupin genome.

**Data S1** Predicted gene family expansions and contractions.

**Data S2** Summary of sequence‐based genetic markers and their mapping to assembled scaffold sequences of narrow‐leafed lupin cv. Tanjil.

**Data S3** An updated genetic map for narrow‐leafed lupin cv. Tanjil.

**Data S4** CEGMA analysis.

**Data S5** CoReFinder analysis.

**Data S6** Dot plot comparisons between narrow‐leafed lupin and other legumes.

**Data S7** Histograms of synonymous‐site changes between paralogous genes between pairs of sequenced legume genomes.

**Data S8** Functional annotations assigned to gene annotations of narrow‐leafed lupin.

**Data S9** References for Table 4.
