# Supplementary material for: A comprehensive draft genome sequence for lupin (Lupinus angustifolius), an emerging health food: insights into plant–microbe interactions and legume evolution
Source: Plant Biotechnol J. 2016 Sep 23;15(3):318–30. doi: 10.1111/pbi.12615 (PMC5316927; doi:10.1111/pbi.12615)
Supplement: Supplementary file 1 — Figure S1 K‐mer‐based estimation of genome size based on frequency analysis of 17‐mers in paired‐end libraries. A primary coverage peak in 17‐mer frequency was observed at 39× coverage, which corresponded to a frequency of 37 098 706 666 and 44 885 658 200 bases. Using the equation (K‐mer frequency/Peak depth) the K‐mer frequency analysis estimated a total genome size of 951 248 889 bp or 951.2 Mb. This estimate is consistent with C‐value based estimates of 924 Mb. Figure S2 Summary of sequence divergence (100% – sequence identity) across DNA transposon and retrotransposon families (LTR, LINE and SINE) predicted in the narrow‐leafed lupin cv. Tanjil genome assembly relative to: (a) a representative repeat sequence in Repbase; or (b) de novo repeat family consensus sequence. Figure S3 Length distributions for gene annotation sub‐feature including mRNA (a), coding sequence (CDS) (b), exons (c) and introns (d). Figure S4 (a) Length (cM) of the 20 linkage groups of the 83A:476 × P27255 RIL genetic map using 2959 non‐redundant molecular markers*. (b) Average interval size (cM) of the 83A:476 × P27255 RIL genetic map using 2959 non‐redundant molecular markers*. *Out of the 9972 markers genotyped in the RIL population (n = 153) 2959 had unique (non‐redundant) centiMorgan positions in the genetic map. Of the 9972 markers 7707 physically mapped unambiguously to unique locations in the pseudomolecule assembly and were used to orientate and assign scaffolds to pseudomolecules. Figure S5 Venn diagram showing shared orthologous groups in narrow‐leafed lupin and other sequenced plant genomes. Figure S6 Protein orthology comparison of narrow‐leafed lupin (L. angustifolius) and other sequenced plant species. Figure S7 Estimation of divergence time for narrow‐leafed lupin (L. angustifolius) and selected other sequenced legume species. Red numbers are estimated divergence times from the present day in millions of years (Mya), derived relative to an assumed time of 58 Mya for the orig [file PBI-15-318-s009.docx]

# Supplementary Tables

**Table S1** Summary of the total amount of sequence data generated for the *L, angustifolius* cv. Tanjil genome assembly and the average coverage per paired-end and mate-pair library, assuming an estimated genome size of 924 Mb based on C value prediction.

| **Library type** | **insert size (bp)** | **total length (Gb)** | **X coverage**  **(of 924 Mb)** |
| --- | --- | --- | --- |
| paired end | 170 | 22.28 | 24.11 |
| paired end | 500 | 30.69 | 33.22 |
| paired end | 800 | 22.98 | 24.87 |
| mate pair | 2,000 | 20.84 | 22.55 |
| mate pair | 5,000 | 11.15 | 12.07 |
| mate pair | 10,000 | 15.09 | 16.33 |
| mate pair | 20,000 | 19.18 | 20.76 |
| mate pair | 40,000 | 8.20 | 8.87 |

Table S2 Summary of repetitive DNA regions predicted within the narrow-leafed lupin genome.

| **Method** | **Repeat Size (bp)** | **% of genome** |
| --- | --- | --- |
| Tandem Repeat Finder | 34,385,328 | 5.63 |
| RepeatMasker | 95,943,148 | 15.71 |
| RepeatProteinMask | 108,715,411 | 17.81 |
| *De novo* | 325,013,936 | 53.24 |
| Total (merged) | 347,149,759 | 56.87 |

Table S3 Summary of non-coding RNA genes predicted within the narrow-leafed lupin cv. Tanjil genome assembly.

|  | **Type** | **Copy** | **Average length(bp)** | **Total length(bp)** | **% of genome** |
| --- | --- | --- | --- | --- | --- |
| miRNA |  | 307 | 124.013 | 38,072 | 0.006 |
| tRNA |  | 1,047 | 74.800 | 78,316 | 0.012 |
| rRNA | rRNA | 1,277 | 167.381 | 213,746 | 0.035 |
|  | 18S | 121 | 723.074 | 87,492 | 0.014 |
|  | 28S | 206 | 127.563 | 26,278 | 0.004 |
|  | 5.8S | 53 | 138.094 | 7,319 | 0.001 |
|  | 5S | 897 | 103.296 | 92,657 | 0.015 |
| snRNA | snRNA | 499 | 115.060 | 57,415 | 0.009 |
|  | CD-box | 315 | 97.901 | 30,839 | 0.005 |
|  | HACA-box | 47 | 122.468 | 5,756 | 0.001 |
|  | splicing | 137 | 151.970 | 20,820 | 0.003 |

Table S4 Summary of proteomics analyses applied to four tissues of narrow-leafed lupin and the number of peptides and proteins identified at a 99% confidence interval.

| **Database** | **Tissue** | **Spectra** | **Peptides** | **Proteins** |
| --- | --- | --- | --- | --- |
| Translated gene annotations | Leaf | 107,182 | 7,246 | 1,134 |
|  | Seed | 15,818 | 1,963 | 276 |
|  | Stem | 2,826 | 679 | 164 |
|  | Root | 17,812 | 3,827 | 657 |
| 6-frame translated scaffolds  (ORFs ≥ 10 aa between stop codons) | Leaf | 83,518 | 4,792 | 529 |
|  | Seed | 12,408 | 1,423 | 190 |
|  | Stem | 1,833 | 348 | 94 |
|  | Root | 11,333 | 1,970 | 374 |

**Table S5** Summary of functional annotations assigned to gene annotations in narrow-leafed lupin cv. Tanjil.

|  | **Number** | **Percent (%)** |
| --- | --- | --- |
| Total | 33,076 |  |
| InterPro | 26,580 | 80.4 |
| GO | 20,142 | 60.9 |
| KEGG | 19,425 | 58.7 |
| Swissprot | 24,890 | 75.3 |
| TrEMBL | 9,667 | 29.2 |
| Annotated | 29,204 | 88.3 |
| Unannotated | 3,872 | 11.7 |

Table S6 Summary statistics for 20 narrow-leafed lupin linkage groups NLL-01 to NLL-20 comprising 9,965 molecular markers and 7 trait loci.

| **Linkage group** | **Framework loci** | **Redundant loci** | **Attached loci** | **Total loci** | **Length (cM)*** | **Average interval size (cM)** ♰ |
| --- | --- | --- | --- | --- | --- | --- |
| NLL-01 | 197 | 343 | 75 | 615 | 180.8 | 0.92 |
| NLL-02 | 125 | 318 | 79 | 522 | 143.7 | 1.16 |
| NLL-03 | 171 | 302 | 66 | 539 | 149.1 | 0.88 |
| NLL-04 | 194 | 277 | 45 | 516 | 148.4 | 0.77 |
| NLL-05 | 138 | 288 | 76 | 502 | 122 | 0.89 |
| NLL-06 | 242 | 462 | 96 | 800 | 179.6 | 0.75 |
| NLL-07 | 146 | 315 | 86 | 547 | 122.4 | 0.84 |
| NLL-08 | 202 | 368 | 83 | 653 | 157.6 | 0.78 |
| NLL-09 | 149 | 198 | 76 | 423 | 127.5 | 0.86 |
| NLL-10 | 106 | 181 | 69 | 356 | 99 | 0.94 |
| NLL-11 | 187 | 364 | 71 | 622 | 147 | 0.79 |
| NLL-12 | 122 | 262 | 60 | 444 | 103.4 | 0.85 |
| NLL-13 | 120 | 247 | 55 | 422 | 108.2 | 0.91 |
| NLL-14 | 98 | 200 | 55 | 353 | 91.8 | 0.95 |
| NLL-15 | 130 | 290 | 73 | 493 | 107.3 | 0.83 |
| NLL-16 | 115 | 231 | 64 | 410 | 99.2 | 0.87 |
| NLL-17 | 131 | 257 | 61 | 449 | 101.1 | 0.78 |
| NLL-18 | 130 | 223 | 53 | 406 | 96.7 | 0.75 |
| NLL-19 | 123 | 211 | 46 | 380 | 105.9 | 0.87 |
| NLL-20 | 133 | 309 | 78 | 520 | 110.1 | 0.83 |
| **TOTAL** | **2959** | **5646** | **1367** | **9972** | **2500.8** | **0.85** |

* Distances presented as Kosambi centi-Morgans (cM)

♰ Between non-redundant framework markers

**Table S7.** Read alignment of narrow-leafed lupin cultivar Tanjil RNASeq data for five different tissue types, paired-end and mate-pair data to the genome.

| Transcriptome tissue type/ Library insert size (bp) | Read pairs | Mapped reads to current assembly | % mapped reads to current assembly |
| --- | --- | --- | --- |
| Root | 13,634,045 | 12,154,536 | 89.1 |
| Leaf | 13,227,168 | 13,049,981 | 98.7 |
| Stem | 12,943,960 | 12,753,223 | 98.5 |
| Seed | 13,625,203 | 13,418,647 | 98.5 |
| Flower | 12,798,135 | 12,663,783 | 99 |
| 170 bp | 161,726,921 | 146,430,351 | 90.5 |
| 500 bp | 228,642,270 | 217,801,684 | 95.3 |
| 800 bp | 172,089,484 | 163,348,967 | 94.9 |
| 2,000 bp | 213,449,190 | 183,488,247 | 86.0 |
| 5,000 bp | 114,259,809 | 96,134,431 | 84.1 |
| 10,000 bp | 154,656,974 | 134,851,592 | 87.2 |
| 20,000 bp | 196,564,496 | 177,865,002 | 90.5 |
| 40,000 bp | 84,039,657 | 76,407,402 | 90.9 |

**Table S8.** Overview of the coverage and variants (insertions/deletions and SNPs) identified for the three re-sequenced narrow-leafed lupin lines.

|  | Unicrop | 83A:476 | P27255 |
| --- | --- | --- | --- |
| Trimmed clean data (Gb) | 51.5 | 59 | 59.2 |
| Coverage (based on 921 Mb genome size determined by flow cytometry) | 62x | 55x | 62x |
| Number of insertions/deletions (indels) | 81,375 | 47,113 | 216,167 |
| Number of single nucleotide polymorphisms (SNPs) | 1,099,966 | 606,035 | 3,053,917 |

**Table S9.** Location of domestication traits and disease resistance genes in the genome assembly of narrow-leafed lupin cv. Tanjil

| Trait | Locus name | LG | Region | Upper marker | Lower marker | Co-segregating marker(s) |
| --- | --- | --- | --- | --- | --- | --- |
| Anthracnose resistance | *Lanr1* | NLL-11 | 388 Kb | LaDArT_PAV20595 | LaDArT_PAV25221 | \| LaDArT_SNP16268 \| \| --- \| \| LaDArT_SNP14923 \| \| LaSNP_022 \| \| LaDArT_SNP14593 \| \| LaDArT_PAV23977 \| \| LaDArT_PAV06982 \| |
|  |  |  |  |  |  |  |
| Phomopsis resistance* | *PhtjR* | NLL-05 | 127.6 Kb | PhtjM2 | PhtjM4 | PhtjM3 |
|  |  |  |  |  |  |  |
| Bitterness locus | *Iucundus* | NLL-07 | 746 Kb | LaSSR_025 | LaSNP_509 | - |
|  |  |  |  |  |  |  |
| Flowering time | *Ku* | NLL-10 | 413 Kb | LaDArT_SNP01240 | LaSNP_449 | dFTc  KuHM1  UWA214  A071b |
|  |  |  |  |  |  |  |
| Pod shattering | *Tardus* | NLL-01 | 517.6 Kb | LaDArT_PAV01153 | LaDArT_PAV26383 | LaDArT_PAV02725  TaM1  IPb-329002 |
|  |  |  |  |  |  |  |
| Pod shattering | *Lentus* | NLL-08 | 387.1 Kb | LaDArT_SNP11596 | LaDArT_PAV14506 | LaDArT_PAV09840  LaDArT_SNP05271  LaDArT_SNP18104  LaDArT_SNP15906  LaDArT_PAV22593  LaDArT_PAV23955 |
|  |  |  |  |  |  |  |
| Soft seededness | *Mollis* | NLL-17 | 119.5 Kb | LaDArT_SNP17669 | LaDArT_SNP16412 | MoA |
|  |  |  |  |  |  |  |
| Flower colour | *Leucospermus* | NLL-03 | 907.1 Kb | LaDArT_SNP00080 | LaDArT_SNP14731 | LaDArT_SNP01006  LaDArT_SNP18537  LaDArT_SNP09012  LaDArT_PAV02765  LaDArT_PAV06411  LaDArT_PAV27455  LaDArT_PAV11801  LaDArT_PAV12744 |

*Molecular markers from Yang et al 2013. Rapid development of molecular markers by next-generation sequencing linked to a gene conferring phomopsis stem blight disease resistance for marker-assisted selection in lupin (Lupinus angustifolius L.) breeding. *Theoretical and Applied Genetics* **126,** 511-522.

Table S10 Summary of orthologous gene families in narrow-leafed lupin and other sequenced plant species.

| **Species** | **Genes number** | **Genes in families** | **Unclustered genes** | **Family number** | **Unique families** | **Average genes per family** |
| --- | --- | --- | --- | --- | --- | --- |
| *L. angustifolius* | 33,076 | 27,885 | 5,191 | 15,257 | 563 | 1.83 |
| *C. cajan* | 39,712 | 32,916 | 6,796 | 18,266 | 1,224 | 1.8 |
| *C. arietinum* | 28,269 | 25,124 | 3,145 | 15,281 | 377 | 1.64 |
| *G. max* | 42,859 | 36,452 | 6,407 | 17,424 | 284 | 2.09 |
| *M. truncatula* | 43,683 | 30,674 | 13,009 | 14,508 | 2,198 | 2.11 |
| *P. vulgaris* | 27,088 | 24,619 | 2,469 | 17,366 | 210 | 1.42 |
| *A. thaliana* | 26,637 | 22,628 | 4,009 | 13,329 | 920 | 1.7 |

**Table S11**. Synteny coverage depth for NLL vs. other genomes and for other genomes vs. NLL. Each row begins with a coverage depth (0, 1, 2, etc.). Percentages for a given coverage depth and a species indicate the proportion of the genome with the indicated synteny coverage depth, with respect to the indicated reference genome. For example, in table 11A, 21.4% of the Lotus genome is covered by three synteny features with NLL.

| A. Percent of NLL genome with indicated synteny depth, with species X as reference | | | | | |
| --- | --- | --- | --- | --- | --- |
|  | *Glycine* | *Lotus* | NLL | *Medicago* | *Phaseolus* |
| 0 | 49.25% | 20.30% | 68.62% | 23.33% | 41.96% |
| 1 | 10.97% | 19.89% | 21.84% | 16.94% | 15.43% |
| 2 | 11.35% | 24.75% | 9.34% | 22.27% | 15.80% |
| 3 | 12.73% | 21.40% | 0.19% | 20.95% | 13.24% |
| 4 | 8.81% | 9.57% | 0.03% | 10.49% | 8.75% |
| 5 | 5.19% | 3.61% |  | 4.49% | 4.00% |
| 6 | 1.58% | 0.47% |  | 1.39% | 0.74% |
| 7 | 0.12% |  |  | 0.09% | 0.08% |
| 8 | 0.00% |  |  | 0.02% |  |
|  |  |  |  |  |  |
| B. Percent of species X genome with indicated synteny depth, with NLL as reference | | | | | |
|  | *Glycine* | *Lotus* | NLL | *Medicago* | *Phaseolus* |
| 0 | 28.44% | 46.96% | 68.62% | 32.80% | 34.74% |
| 1 | 7.84% | 38.08% | 21.84% | 38.55% | 34.74% |
| 2 | 26.33% | 14.19% | 9.34% | 27.48% | 28.57% |
| 3 | 12.56% | 0.62% | 0.19% | 1.08% | 1.73% |
| 4 | 22.93% | 0.13% | 0.03% | 0.06% | 0.20% |
| 5 | 0.81% | 0.01% |  | 0.04% | 0.02% |
| 6 | 0.83% | 0.00% |  |  |  |
| 7 | 0.18% |  |  |  |  |
| 8 | 0.07% |  |  |  |  |

**Table S12**. Values under "WGD Ks peaks" are the Ks bin values for the mode in Ks plots from the indicated species pairs, corresponding to the papilionoid whole-genome duplication (WGD) in that Ks plot. Plots can be seen in Figure 2 and in Supplemental Data File 5. For example, for *Glycine*-*Glycine*, this would be the second modal peak in the orthologous plot, Figure 2B (the most recent peak being the one from the WGD within the *Glycine* lineage). For *Glycine*-*Lupinus*, the WGD peak is older than the speciation peak. This can be seen in Figure 2A, second asterisk. Values under "Speciation Ks peaks" are also inferred from Figure 2 and in Supplemental Data File 6. For example, for *Glycine*-*Lupinus*, the speciation peak can be seen in Figure 2A, first asterisk. Values under "Recent independent WGD peaks" are seen in Figure 2B for *Glycine*-*Glycine* and *Lupinus*-*Lupinus*.

| **WGD Ks peaks** | *Glycine* | *Lupinus* | *Lotus* | *Medicago* | *Phaseolus* |
| --- | --- | --- | --- | --- | --- |
| *Glycine* | 0.68 | 0.68 | 0.7 | 0.88 | 0.76 |
| *Lupinus* |  | 0.73 | 0.7 | 0.88 | 0.8 |
| *Lotus* |  |  | 0.62 | 0.8 | 0.72 |
| *Medicago* |  |  |  | 0.96 | 0.94 |
| *Phaseolus* |  |  |  |  | 0.82 |
|  |  |  |  |  |  |
| **Speciation Ks peaks** | *Glycine* | *Lupinus* | *Lotus* | *Medicago* | *Phaseolus* |
| *Glycine* |  | 0.56 | 0.48 | 0.66 | 0.28 |
| *Lupinus* |  |  | 0.6 | 0.7 | 0.62 |
| *Lotus* |  |  |  | 0.6 | 0.56 |
| *Medicago* |  |  |  |  | 0.72 |
| *Phaseolus* |  |  |  |  |  |
|  |  |  |  |  |  |
| **Recent independent WGD peaks** |  |  |  |  |  |
| Gm.x.Gm | 0.13 | 0.065 |  |  |  |
| NLL.x.NLL | 0.31 |  |  |  |  |

**Table S13** Summary of genes and gene families prominent in flowering time control and light signalling in other dicot species present or absent in the narrow-leafed lupin genome.

| **Functional role** | **Gene** | **Encoded protein** | ***Medicago truncatula* ID** | ***Lupinus angustifolius* ID** |
| --- | --- | --- | --- | --- |
| photoreceptor | *PHYA* | red/far-red light photoreceptor | *1g085160* | *Lup017016, Lup002691, Lup015476* |
|  | *PHYB* |  | *2g034040* | *Lup014358, Lup027042* |
|  | *PHYE* |  | *2g049520* | not present |
|  | *CRY1* | blue light photoreceptor | *5g063920* | *Lup004976, Lup002802, Lup012646* |
|  | *CRY2* |  | *1g043180, 1g076190* | *Lup008367* |
|  | *ZTL* | blue light photoreceptor | *2g036510* | *Lup021384, Lup007315* |
|  | *FKF1* |  | *8g105590* | *Lup031570* |
|  | *PHOT1* | blue light photoreceptor | *4g061610, 2g095980* | *Lup011301* |
|  | *PHOT2* |  | *8g070530* | *Lup028173, Lup016543* |
|  | *UVR8* | UV photoreceptor | *3g096780* | *Lup003585, Lup025974, Lup031272* |
| light signalling | *COP1* | ubiquitin ligase component | *5g085250* | *Lup000696* |
|  | *SPA1* | ubiquitin ligase component | *5g009530, 8g027985* | *Lup005516, Lup032080, Lup004875* |
|  | *SPA4* |  | *8g091170, 2g084980, 2g085210* | *Lup023005* |
|  | *HY5* | BZIP transcription factor | *3g436010* | *Lup008440* |
|  | *HYH* |  | *7g057160* | *Lup028646* |
|  | *PIF1* | bHLH transcription factor | *7g099540, 1g069155* | *Lup028329* |
|  | *PIF3* |  | *1g084980, 7g111320* | *Lup015480* |
|  | *PIF4/5* |  | *3g449770* | *Lup019016* |
|  | *PIF6* |  | *7g110810* | *Lup005332* |
|  | *SPT* |  | *5g017040* | *Lup006191, Lup001007, Lup008225?* |
| circadian clock | *LHY* | MYB transcription factor | *7g118630* | *Lup021567* |
|  | *TOC1a/b* | pseudo-response-regulator | *4g108880, 3g037390* | *Lup026326, Lup010052, Lup010053* |
|  | *ELF3a* |  | *3g103970* | *Lup030650* |
|  | *ELF3b* |  | *1g016920* | *Lup000361* |
|  | *LUX* | MYB transcription factor | *4g064730* | *Lup004491, Lup029750* |
|  | *ELF4* |  | *3g070490* | *Lup016731* |
|  | *ELF4-*like |  | *3g070490, 4g125590, 2g041310* | *Lup020202, Lup026070* |
|  | *PRR59a* | pseudo-response-regulator | *3g092780* | *Lup009602, Lup000501* |
|  | *PRR59b/c* |  | *7g118260, 8g024260* | *Lup002529, Lup008505* |
|  | *PRR37a* |  | *4g061360* | *Lup017167* |
|  | *PRR37b* |  | *1g067110* | *Lup018664* |
|  | *GI* |  | *1g098160* | *Lup002034, Lup004730, Lup025070* |
| flowering integration | *FTa1/2* | phosphatidylethanolamine binding-protein | *7g084970, 7g085020* | not present |
|  | *FTa3* |  | *6g033040* | *Lup021189* |
|  | *FTb* |  | *7g066630, 7g066690* | not present |
|  | *FTc* |  | *7g085040* | *Lup015264, Lup005674* |
|  | *TFL1* |  | *1g060190, 2g086270, 7g104460* | *Lup019608, Lup026028, Lup001307, Lup025777,* |
|  | *MFT* |  | *8g106840* | *Lup021954* |
|  | *BFT* |  | *0020s120* | *Lup020111* |
|  | *FDa* | BZIP transcription factor | *5g022780* | *Lup018024* |
|  | *FDb* |  | *8g075130* | not present |
|  | *LFY* |  | *3g098560* | *Lup027481a, Lup006312, Lup012198* |
| photoperiod response | *COLa* | B-box transcription factor | *7g018170* | *Lup011967, Lup012039, Lup001378* |
|  | *COLb/c* |  | *1g013450, 3g015710* | *Lup009876, Lup030682* |
|  | *COLd* |  | *4g128930* | *Lup009465, Lup026154* |
|  | *COLe/f* |  | *3g082630, 5g069480* | *Lup032539, Lup032195, Lup028850, Lup022494, Lup025079* |
|  | *COLg* |  | *7g108350* | *Lup006262, Lup002871* |
|  | *COLh* |  | *7g083540* | not present |
|  | *COLj* |  | *2g088900* | *Lup006522* |
|  | *COLi* |  | *8g104190* | *Lup029233, Lup032792, Lup011055* |
|  | *COLk* |  | *1g110870* | *Lup016892, Lup018321, Lup030499* |
|  | *CDFa* | DOF transcription factor | *3g435480* | *Lup015794, Lup018442* |
|  | *CDFb* |  | *7g010950, 6g012450* | *Lup004204, Lup011979* |
|  | *CDFc* |  | *4g082060, 5g014530* | *Lup006734, Lup008651* |
|  | *CDFd* |  | *8g044220, 7g086780* | *Lup001785* |
| MADS | *FULa/b* | MADS domain transcription factor | *4g109830, 2g461760* | *Lup018485* |
|  | *FULc* |  | *7g016630* | *Lup023253, Lup029962* |
|  | *AP1* |  | *8g066260, 5g046790* | *Lup021855, Lup024348, Lup006876* |
|  | *SEP1/2* |  | *7g016600, 6g015975* | *Lup001397, Lup029963* |
|  | *SEP3/4* |  | *8g097090, 3g084980, 4g109810* | *Lup007613, Lup024347, Lup021854, Lup026304, Lup018484* |
|  | *SOC1* |  | *7g075870, 8g033250* | *Lup024911, Lup014751* |
|  | *AGL19/14* |  | *4g102530* | *Lup020546* |
|  | *AGL71/72* |  | *not present* | not present |
|  | *SHP* |  | *3g452380* | *Lup029632, Lup019040* |
|  | *AG* |  | *2g017865, 8g087860* | *Lup005280, Lup011389* |
|  | *STK* |  | *3g005530* | *Lup017516* |
|  | *FLC* clade |  | *not present* | not present |
|  | *ANR1* clade | | *5g031000, 2g009890* | *Lup009010, Lup020683* |
|  | *AGL6/13* |  | *7g075850, 8g033270* | *Lup014750, Lup011089* |
|  | *SVPa* |  | *5g032150, 5g032520, 4g093970* | *Lup015985, Lup025549* |
|  | *SVPc* |  | *5g066180* | *Lup032504, Lup012854* |
|  | *AGL18* |  | *1g053070* | *Lup016817* |
|  | *AGL15* |  | *0003s0590* | *Lup017483, Lup015658* |
|  | *AP3* |  | *5g021270, 3g113030* | *Lup022019, Lup022831* |
|  | *PI* |  | *3g088615, 1g029670* | *Lup011754, Lup011756, Lup019933* |
|  | *TT16* |  | *1g038300* | *Lup018771, Lup010795* |
| miscellaneous flowering | *VIN3* | PHD-type zinc finger transcription factor | *4g127880* | *Lup009440, Lup026125, Lup013437* |
|  | *FRI* | – | *3g098290* | *Lup000206* |
|  | *FRLa/b* | – | *4g461760, 3g056070* | *Lup019103* |
|  | *AP2* | AP2-domain transcription factor | *5g016810, 7g100590* | *Lup023235, Lup006193, Lup008869* |
|  | *AP2-*like |  | *1g049140, 6g033255* | *Lup028300* |
|  | *TOE1* |  | *4g061200, 2g093060* | *Lup030221* |
|  | *SNZ/SMZ* |  | *not present* | not present |
|  | *E1* | RAV-domain transcription factor | *2g058520* | *Lup021394, Lup021264* |
|  | *TEM* | RAV-domain transcription factor | *1g093600, 5g053920* | *Lup007788, Lup019206* |

# Supplementary Figures

**Figure S1** K-mer-based estimation of genome size based on frequency analysis of 17-mers in paired-end libraries. A primary coverage peak in 17-mer frequency was observed at 39X coverage, which corresponded to a frequency of 37,098,706,666 and 44,885,658,200 bases. Using the equation (K-mer frequency / Peak depth) the K-mer frequency analysis estimated a total genome size of 951,248,889 bp or 951.2 Mb. This estimate is consistent with C-value based estimates of 924 Mb.


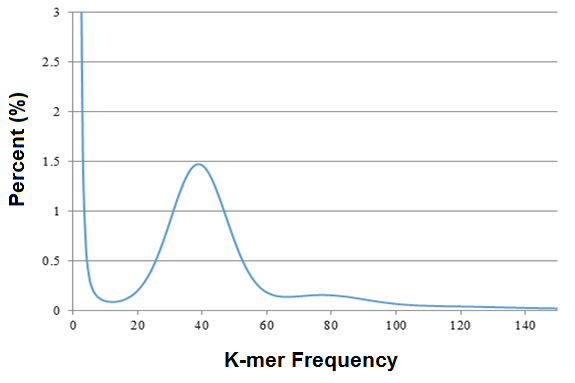


Figure S2 Summary of sequence divergence (100% - sequence identity) across DNA transposon and retrotransposon families (LTR, LINE and SINE) predicted in the narrow-leafed lupin cv. Tanjil genome assembly relative to: A) a representative repeat sequence in REPBASE; or B) *de novo* repeat family consensus sequence.


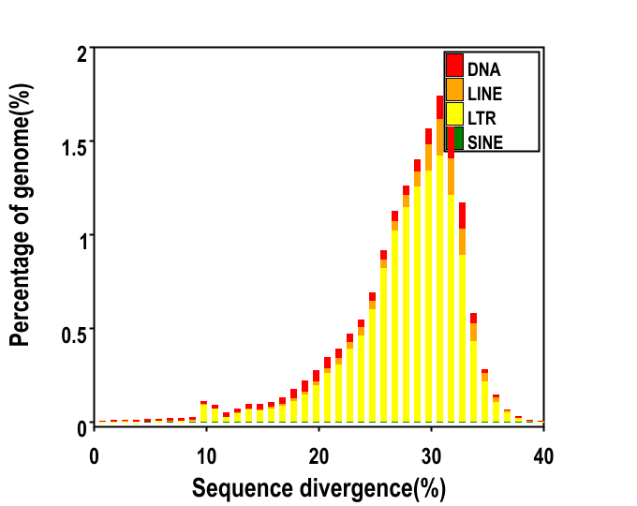

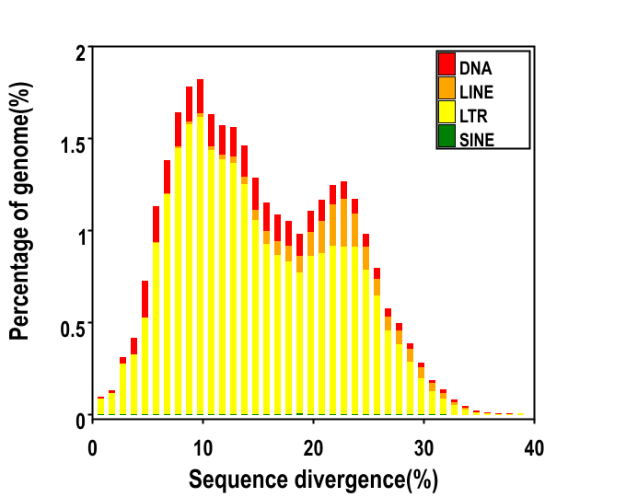


Figure S3 Length distributions for gene annotation sub-feature including mRNA (A), coding sequence (CDS) (B), exons (C) and introns (D).


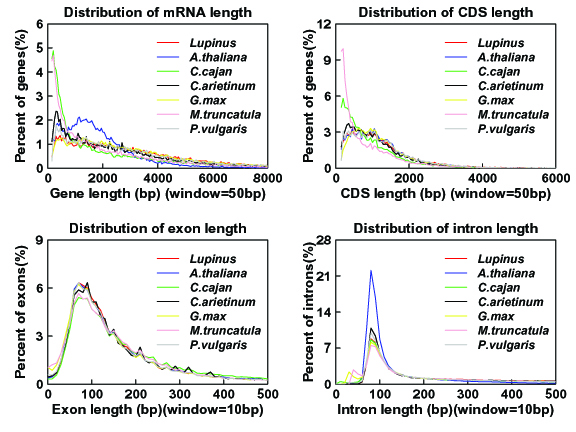


Figure S4 a) Length (cM) of the 20 linkage groups of the 83A:476 x P27255 RIL genetic map using 2,959 non-redundant molecular markers*. b) Average interval size (cM) of the 83A:476 x P27255 RIL genetic map using 2,959 non-redundant molecular markers*. *Out of the 9,972 markers genotyped in the RIL population (n=153) 2,959 had unique (non-redundant) centiMorgan positions in the genetic map. Of the 9,972 markers 7,707 physically mapped unambiguously to unique locations in the pseudomolecule assembly and were used to orientate and assign scaffolds to pseudomolecules.

Figure S5 Venn diagram showing shared orthologous groups in narrow-leafed lupin and other sequenced plant genomes.


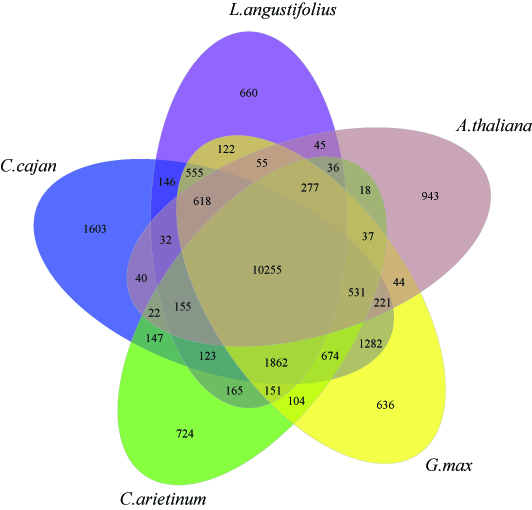


Figure S6 Protein orthology comparison of narrow-leafed lupin (*L. angustifolius*) and other sequenced plant species.


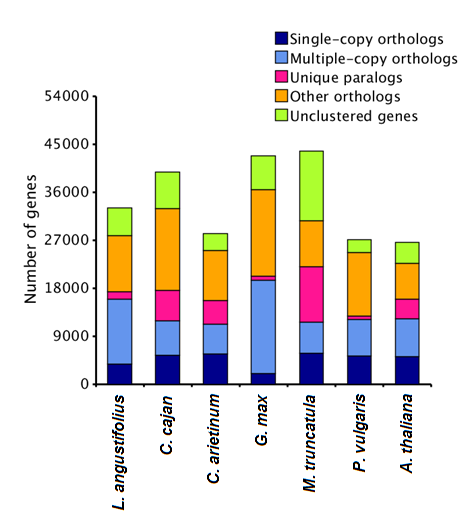


Figure S7 Estimation of divergence time for narrow-leafed lupin (*L. angustifolius*) and selected other sequenced legume species. Red numbers are estimated divergence times from the present day in millions of years (Mya), derived relative to an assumed time of 58 Mya for the origin of the papilionoid clade and papilionoid whole-genome duplication at effectively the same time. Black numbers on the branches are rates of synonymous-site changes (Ks), calculated from modal Ks values between all paralogous genes between the species included above (Data S6). Asterisks mark whole-genome duplications/triplication.

Figure S8 Alignment of translated amino-acid sequences corresponding to narrow-leafed lupin cv. Tanjil locus *Lup005042.1* for anthracnose-resistant (*Colletotrichum lupini*) NLL lines 83A:476 and Tanjil and susceptible NLL lines Unicrop and P27255. The translated amino-acid sequences for 83A:476, P27255 and Unicrop were derived from the re-sequencing data for these accessions/cultivars.


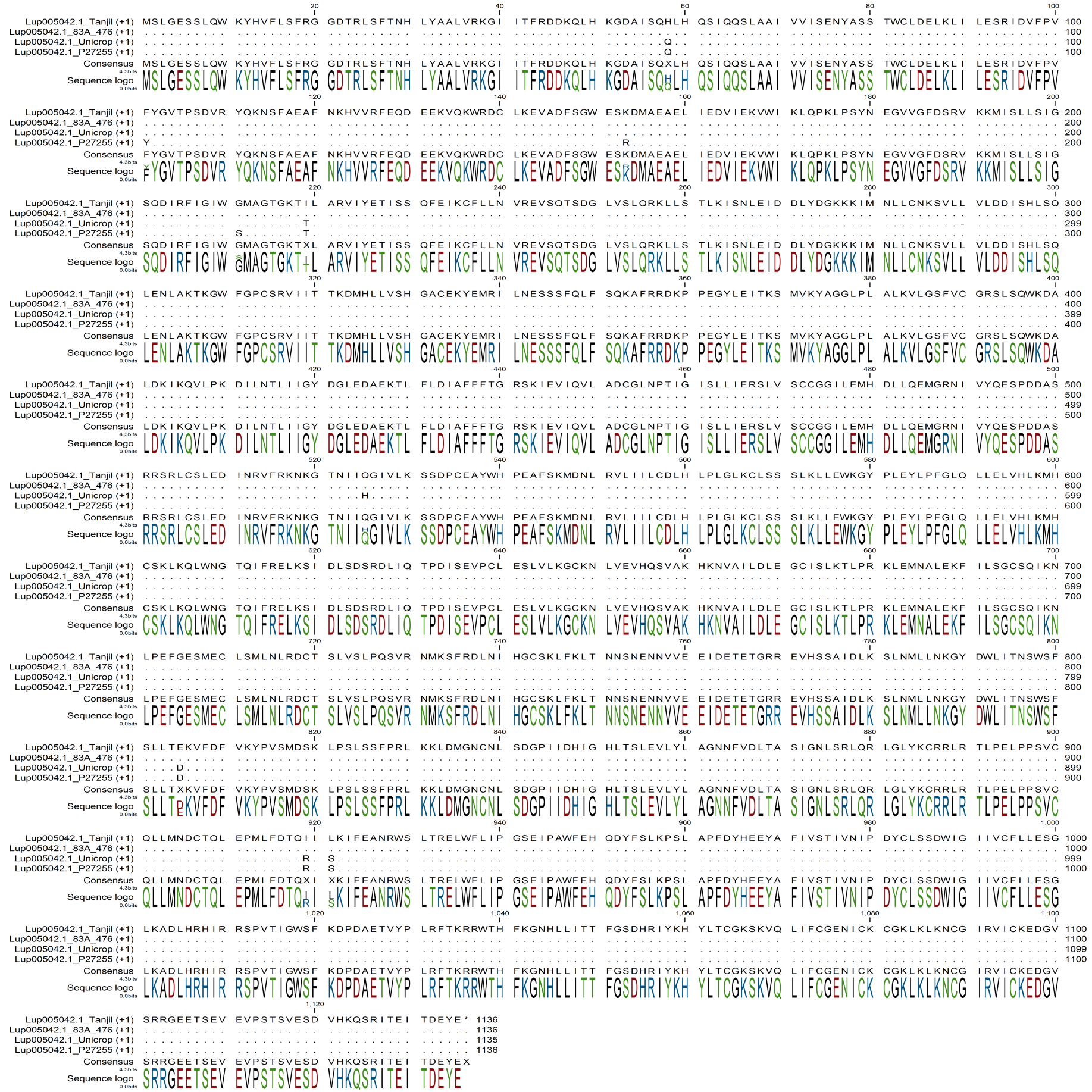


Figure S9 Schematic representation of microsynteny analysis of the *FTa*, *FTb*, and *FTc* gene clusters in barrel medic (*M. truncatula*), chickpea (*C. arietinum*), common bean (*Phaseolus vulgaris*), soybean (*G. max*) and narrow-leafed lupin (*L. angustifolius*) showing that *FTa1, FTa2* and *FTb* genes are absent in these microsyntenic regions of NLL. A) *FTb* region microsynteny B) *FTa1/2* and *FTc1/2* region microsynteny; C) *FTa3/4* region microsynteny.


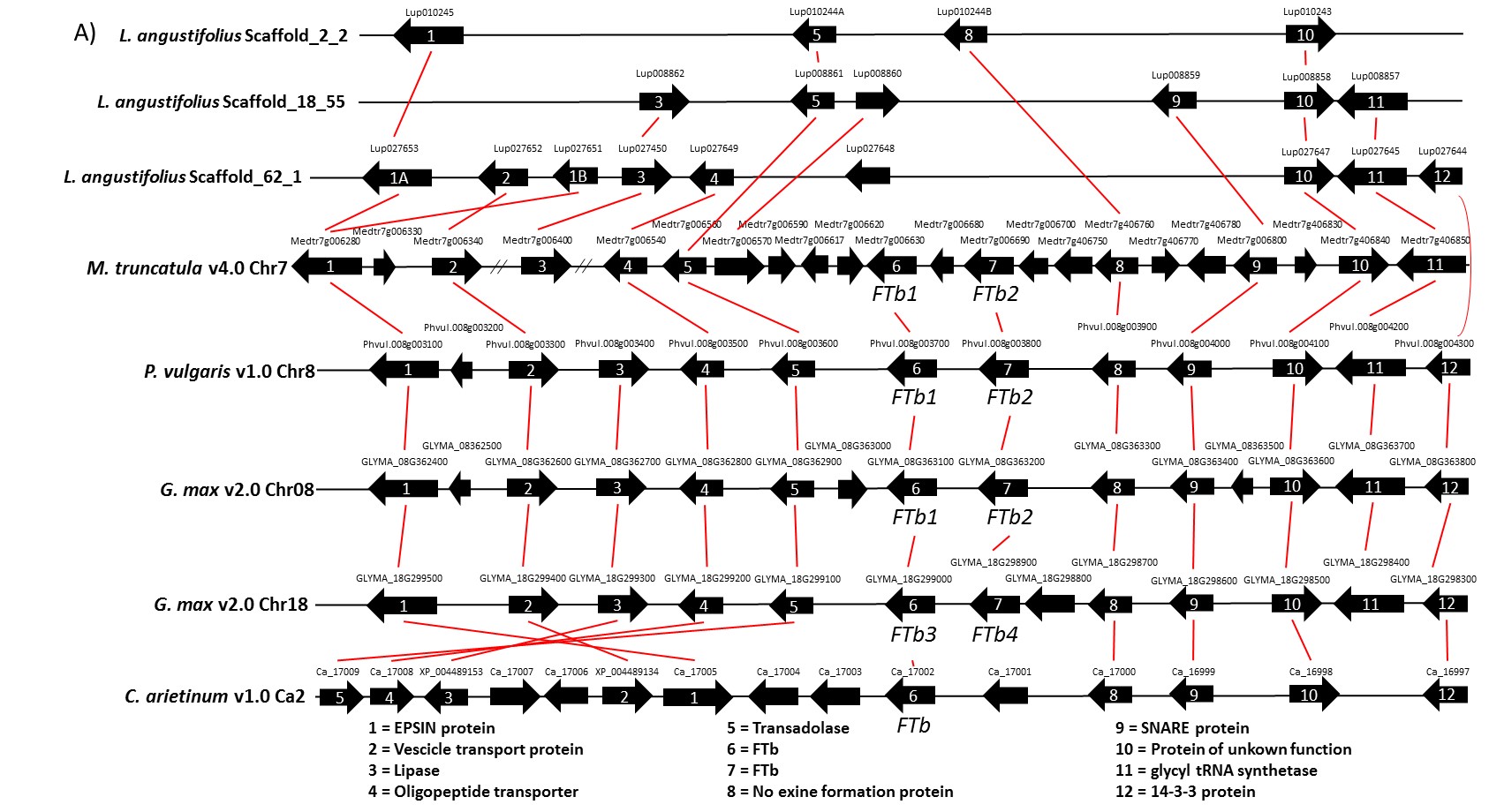


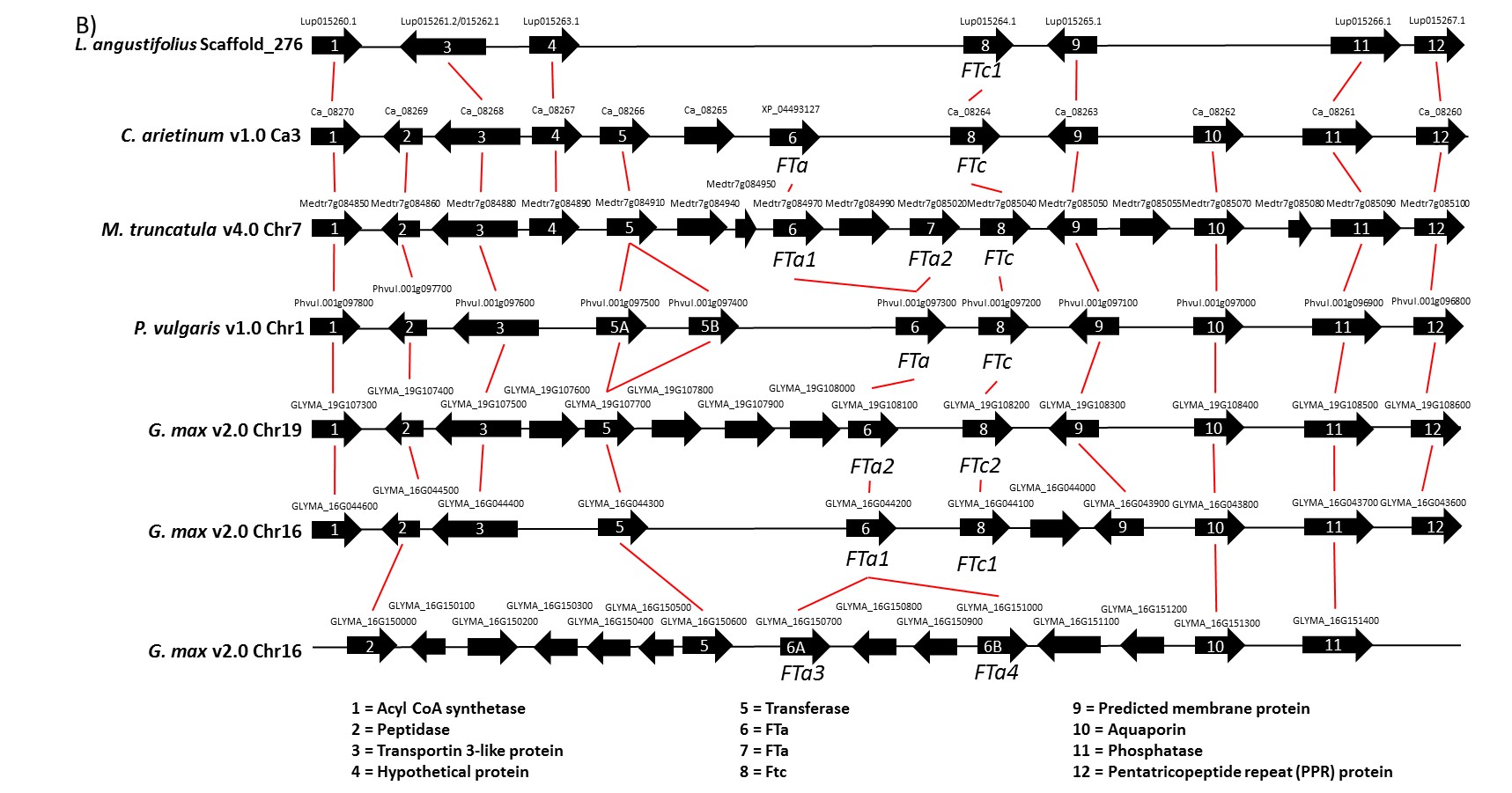


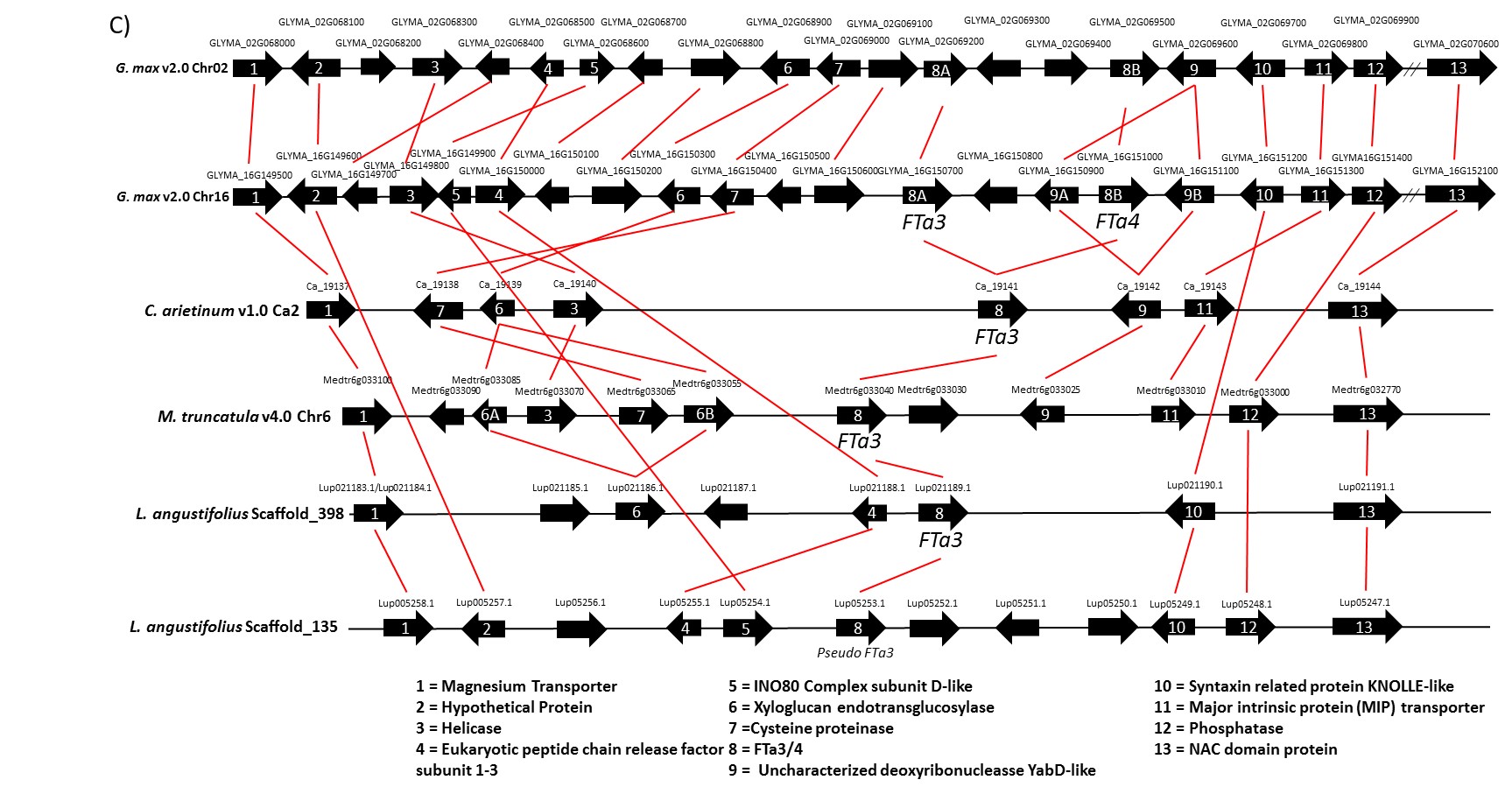


# Supplementary Data Files

Data S1 Predicted gene family expansions and contractions

Data S2 Summary of sequence-based genetic markers and their mapping to assembled scaffold sequences of narrow-leafed lupin cv. Tanjil.

Data S3 An updated genetic map for narrow-leafed lupin cv. Tanjil.

Data S4 CEGMA analysis.

Data S5 CoReFinder analysis.

Data S6 Dot plot comparisons between narrow-leafed lupin and other legumes.

Data S7 Histograms of synonymous-site changes between paralogous genes between pairs of sequenced legume genomes.

Data S8 Functional annotations assigned to gene annotations of narrow-leafed lupin.

Data S9 References for Table 4.
