# Supplementary figures and images for: A comprehensive draft genome sequence for lupin (Lupinus angustifolius), an emerging health food: insights into plant–microbe interactions and legume evolution
Source: Plant Biotechnol J. 2016 Sep 23;15(3):318–30. doi: 10.1111/pbi.12615 (PMC5316927; doi:10.1111/pbi.12615)

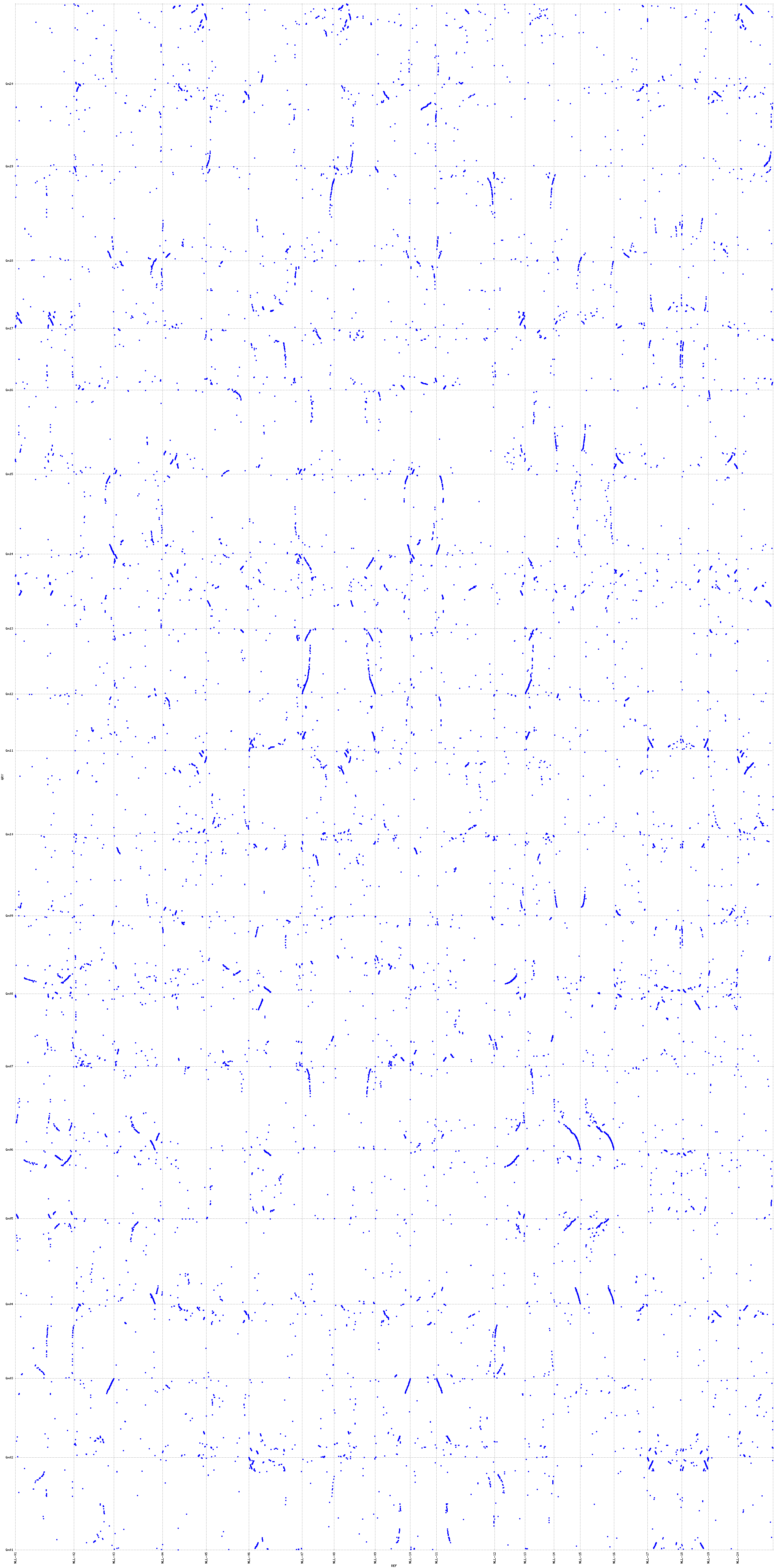

Supplement: Supplementary file 7 — Data S6 Dot plot comparisons between narrow‐leafed lupin and other legumes. [file PBI-15-318-s003.gz › Supp_data_7_dot_plots/NLL_x_glyma.png]

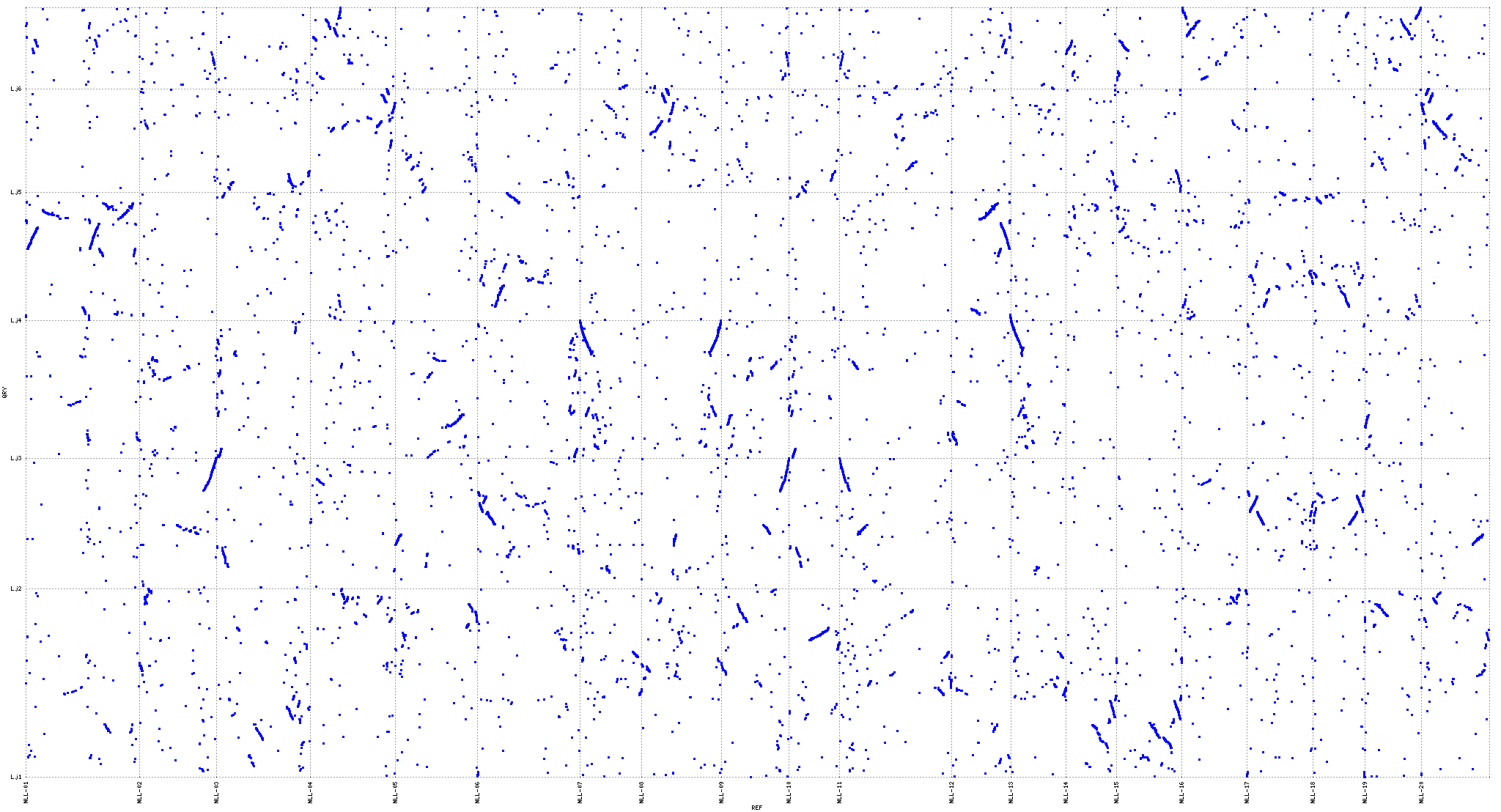

Supplement: Supplementary file 7 — Data S6 Dot plot comparisons between narrow‐leafed lupin and other legumes. [file PBI-15-318-s003.gz › Supp_data_7_dot_plots/NLL_x_lotja.png]

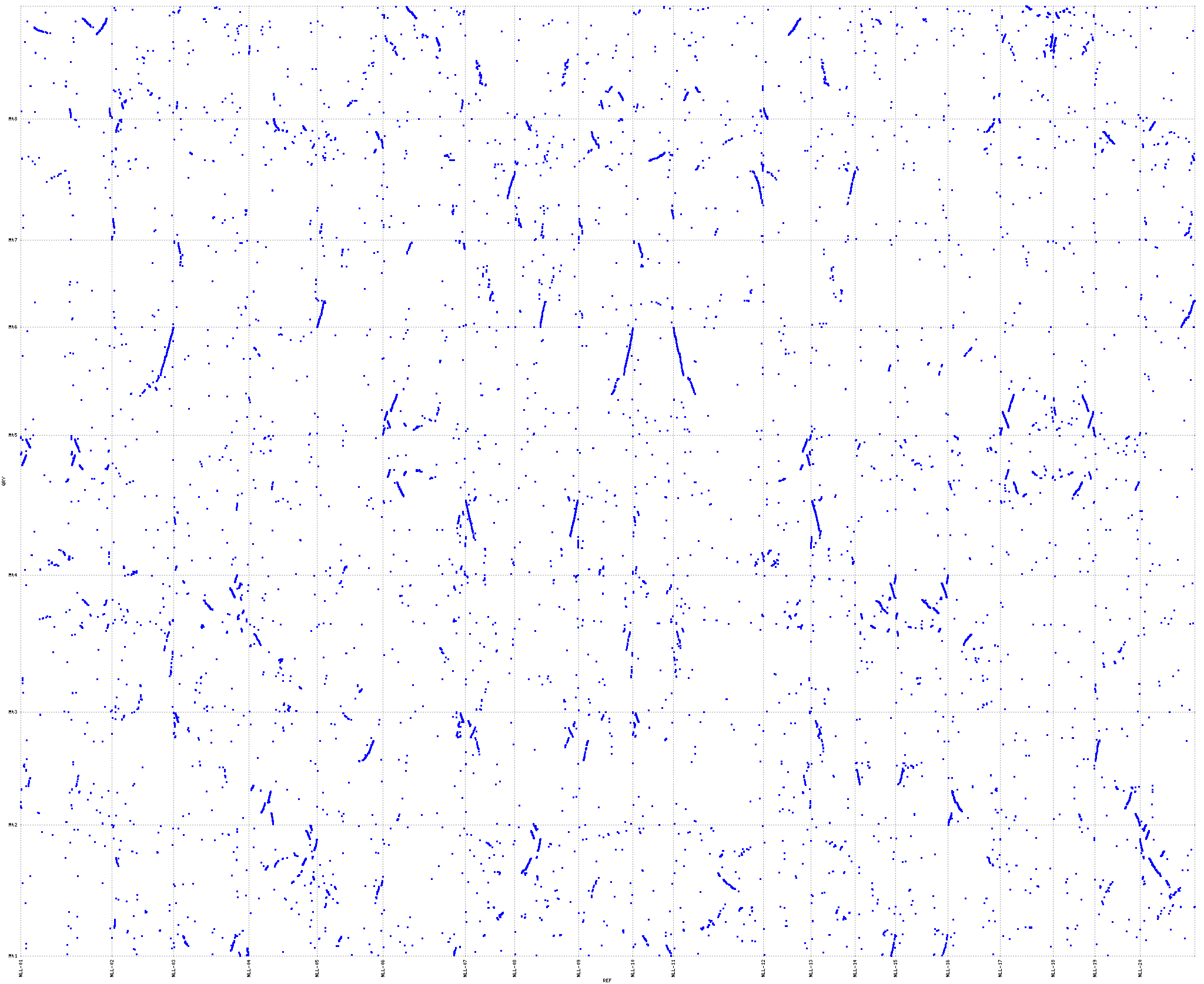

Supplement: Supplementary file 7 — Data S6 Dot plot comparisons between narrow‐leafed lupin and other legumes. [file PBI-15-318-s003.gz › Supp_data_7_dot_plots/NLL_x_medtr.png]

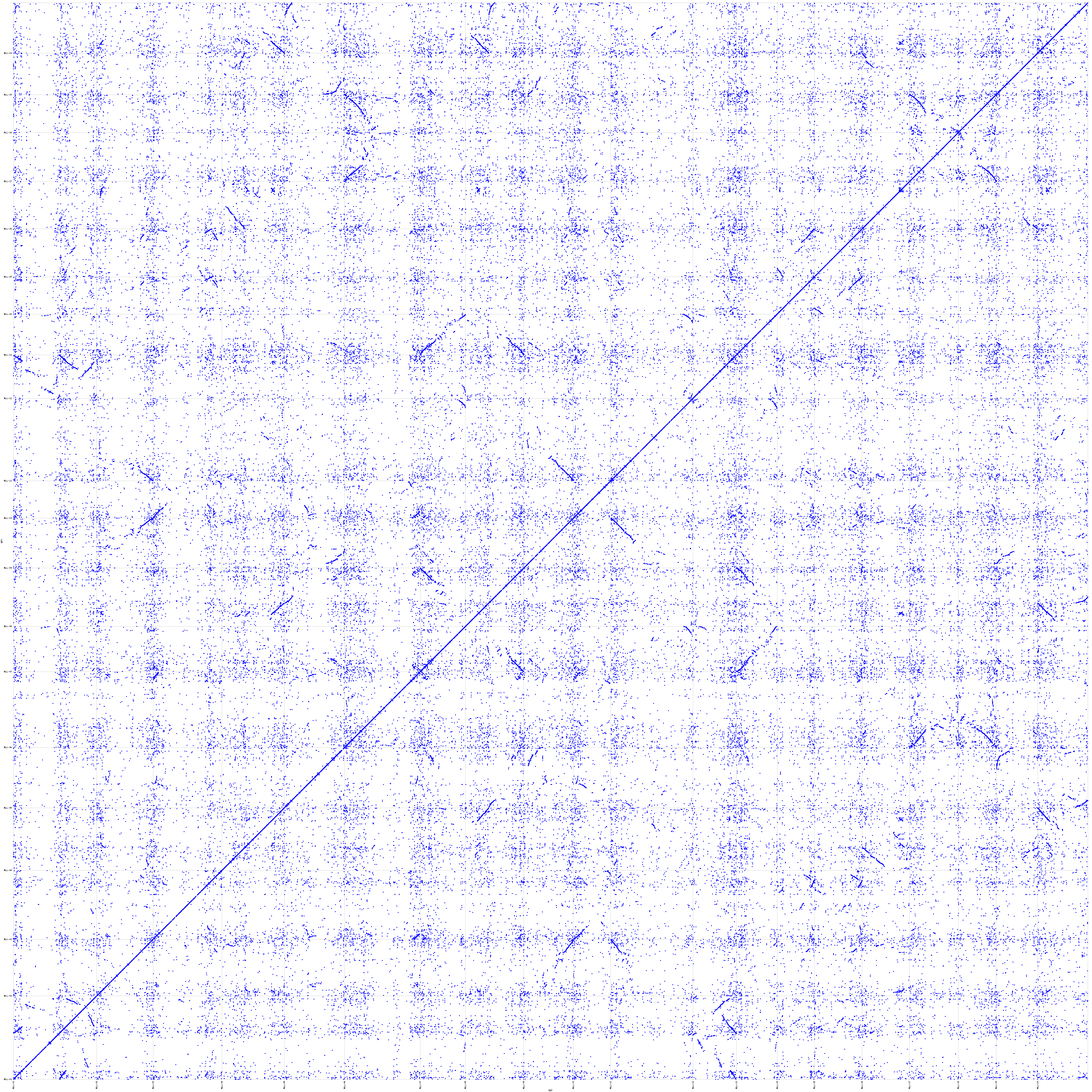

Supplement: Supplementary file 7 — Data S6 Dot plot comparisons between narrow‐leafed lupin and other legumes. [file PBI-15-318-s003.gz › Supp_data_7_dot_plots/NLL_x_NLL.png]

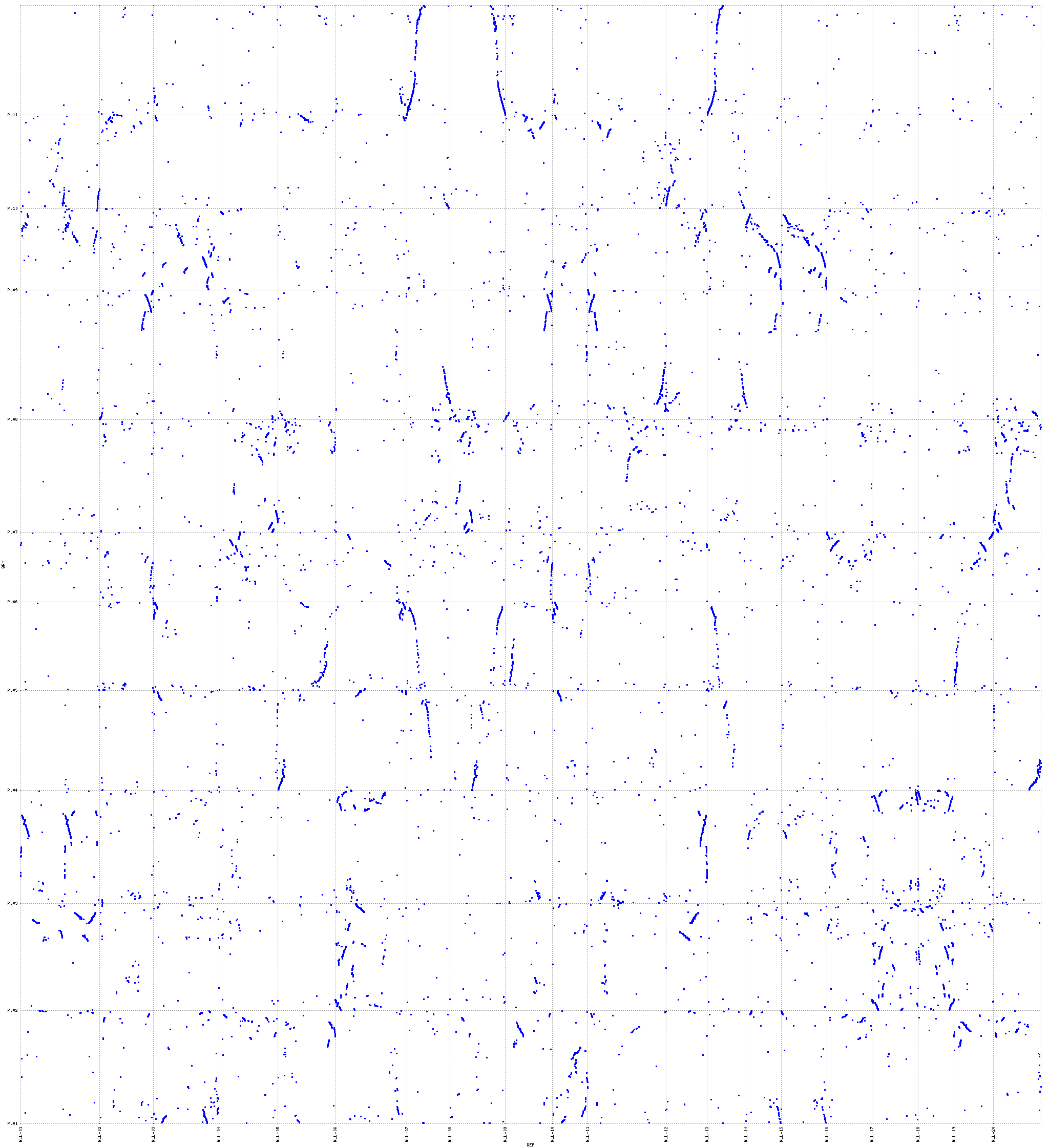

Supplement: Supplementary file 7 — Data S6 Dot plot comparisons between narrow‐leafed lupin and other legumes. [file PBI-15-318-s003.gz › Supp_data_7_dot_plots/NLL_x_phavu.png]
