# Supplementary material for: A comprehensive draft genome sequence for lupin (Lupinus angustifolius), an emerging health food: insights into plant–microbe interactions and legume evolution
Source: Plant Biotechnol J. 2016 Sep 23;15(3):318–30. doi: 10.1111/pbi.12615 (PMC5316927; doi:10.1111/pbi.12615)
Supplement: Supplementary file 10 — Data S9 References for Table 4. [file PBI-15-318-s006.docx]

**Supplementary Data 10** References for Table 4.

1. Saito, K. *et al.* NUCLEOPORIN85 is required for calcium spiking, fungal and bacterial symbioses, and seed production in Lotus japonicus. *Plant Cell* **19,** 610–24 (2007).

2. Kanamori, N. *et al.* A nucleoporin is required for induction of Ca2+ spiking in legume nodule development and essential for rhizobial and fungal symbiosis. *Proc. Natl. Acad. Sci. U. S. A.* **103,** 359–64 (2006).

3. Groth, M. *et al.* NENA, a Lotus japonicus homolog of Sec13, is required for rhizodermal infection by arbuscular mycorrhiza fungi and rhizobia but dispensable for cortical endosymbiotic development. *Plant Cell* **22,** 2509–26 (2010).

4. Capoen, W. *et al.* Nuclear membranes control symbiotic calcium signaling of legumes. *Proc. Natl. Acad. Sci. U. S. A.* **108,** (2011).

5. Floss, D. S., Levy, J. G., Lévesque-Tremblay, V., Pumplin, N. & Harrison, M. J. DELLA proteins regulate arbuscule formation in arbuscular mycorrhizal symbiosis. *Proc. Natl. Acad. Sci. U. S. A.* **110,** E5025–34 (2013).

6. Gomez-Roldan, V. *et al.* Strigolactone inhibition of shoot branching. *Nature* **455,** 189–94 (2008).

7. Kretzschmar, T. *et al.* A petunia ABC protein controls strigolactone-dependent symbiotic signalling and branching. *Nature* 3–8 (2012). doi:10.1038/nature10873

8. van Zeil, A. *et al.* The strigalactone biosynthesis gene DWARDF27 is co-opted in rhizobium symbiosis. *BMC Plant Biol* **15,** 260 (2015).

9. Bitterlich, M., Krügel, U., Boldt-Burisch, K., Franken, P. & Kühn, C. The sucrose transporter SlSUT2 from tomato interacts with brassinosteroid functioning and affects arbuscular mycorrhiza formation. *Plant J.* **78,** 877–89 (2014).

10. Ané, J.-M. *et al.* Medicago truncatula DMI1 required for bacterial and fungal symbioses in legumes. *Science* **303,** 1364–7 (2004).

11. Delaux, P.-M., Bécard, G. & Combier, J.-P. NSP1 is a component of the Myc signaling pathway. *New Phytol.* **199,** 59–65 (2013).

12. Maillet, F. *et al.* Fungal lipochitooligosaccharide symbiotic signals in arbuscular mycorrhiza. *Nature* **469,** 58–63 (2011).

13. Lévy, J. *et al.* A putative Ca2+ and calmodulin-dependent protein kinase required for bacterial and fungal symbioses. *Science* **303,** 1361–4 (2004).

14. Endre, G. *et al.* A receptor kinase gene regulating symbiotic nodule development. *Nature* **417,** 962–6 (2002).

15. Bonfante, P. *et al.* The Lotus japonicus LjSym4 gene is required for the successful symbiotic infection of root epidermal cells. *Mol. Plant. Microbe. Interact.* **13,** 1109–20 (2000).

16. Pumplin, N. *et al.* Medicago truncatula Vapyrin is a novel protein required for arbuscular mycorrhizal symbiosis. *Plant J.* **61,** 482–94 (2010).

17. Yano, K. *et al.* CYCLOPS, a mediator of symbiotic intracellular accommodation. *Proc. Natl. Acad. Sci. U. S. A.* **105,** 20540–5 (2008).

18. Arrighi, J.-F. et al. The Medicago truncatula lysin motif-receptor-like kinase gene family includes NFP and new nodule-expressed genes. *Plant Physiol* **142,** 265–279 (2006).

19. Charpentier, M., Sun, J., Wen, J., Mysore, K. S. & Oldroyd, G. E. D. ABA promotion of arbuscular mycorrhizal colonization requires a component of the PP2A Protein Phosphatase Complex. *Plant Physiol.* **166,** 2077–2090 (2014).

20. **Limpens, E. *et al.*** (2003) LysM domain receptor kinases regulating rhizobial Nod factor-induced infection. *Science* **302,** 630–633 (2003).

21. Middleton, P. H. *et al.* An ERF transcription factor in *Medicago truncatula* that is essential for Nod factor signal transduction. *Plant Cell* **19,** 1221–1234 (2007).

22. Cerri, M. R. *et al****.*** *Medicago truncatula* ERN transcription factors: regulatory interplay with NSP1/NSP2 GRAS factors and expression dynamics throughout rhizobial infection. *Plant Physiol.* **160**, 2155–2172 (2012).

23. Marsh, J. F. *et al.* *Medicago truncatula* NIN is essential for rhizobial-independent nodule organogenesis induced by autoactive calcium/calmodulin-dependent protein kinase. *Plant Physiol.* **144**, 324–335 (2007).

24. Combier, J.-P. *et al.* MtHAP2-1 is a key transcriptional regulator of symbiotic nodule development regulated by microRNA169 in Medicago truncatula. *Genes and Development* **20,** 3084–3088 (2006).

25. Laloum, T., De Mita, S., Games, P., Baudin, M. & Niebel, A. 2013. CCAAT-box binding transcription factors in plants: Y so many? *Trends in Plant Sci* **18,** 157–166 (2012).

26**.** Arrighi, J. F. *et al.* The RPG gene of *Medicago truncatula* controls Rhizobium-directed polar growth during infection. *Proc Natl Acad Sci USA* **105,** 9817-9822 (2008).

27. Kiss, E. *et al.* LIN, a novel type of U-box/WD40 protein, controls early infection by rhizobia in legumes. *Plant Physiol* **151,** 1239–1249 (2009).

28. Mbengue, M. *et al.* The *Medicago truncatula* E3 ubiquitin ligase PUB1 interacts with the LYK3 symbiotic receptor and negatively regulates infection and nodulation. *Plant Cell* **22**, 3474–3488 (2010).

29. Schnabel, E., Journet, E. P., de Carvalho-Niebel, F., Duc , G. & Frugoli, J. The *Medicago truncatula* SUNN gene encodes a CLV1-like leucine-rich repeat receptor kinase that regulates nodule number and root length. *Plant Mol Biol* **58,** 809–22 (2005).

30. Xie, F. *et al.* Legume pectate lyase required for root infection by rhizobia. *Proc Natl Acad Sci USA,* **109,** 633–638 (2012).

31**.** Gonzalez-Rizzo, S., Crespi, M. & Frugier, F**.** (2006) The *Medicago truncatula* CRE1 cytokinin receptor regulates lateral root development and early symbiotic interaction with *Sinorhizobium meliloti*. *Plant Cell* **18,** 2680–93 (2006).

32. Haney, C. H., and Long, S. R. Plant flotillins are required for infection by nitrogen-fixing bacteria. *Proc. Natl. Acad. Sci. U.S.A.* **107,** 478-483 (2010).

33. Pan, H. *et al.* A symbiotic SNARE protein generated by alternative termination of transcription. *Nat. Plants* **2,** 15197 (2016).

34. Takeda, N., Sato, S., Asamizu, E., Tabata, S. & Parniske, M. Apoplastic plant subtilases support arbuscular mycorrhiza development in Lotus japonicus. *Plant J.* **58,** 766–77 (2009).

35. Krajinski, F. *et al.* The H+-ATPase HA1 of Medicago truncatula Is Essential for Phosphate Transport and Plant Growth during Arbuscular Mycorrhizal Symbiosis. *Plant Cell* **26,** 1808–1817 (2014).

36. Wang, E. *et al.* A H+-ATPase That Energizes Nutrient Uptake during Mycorrhizal Symbioses in Rice and Medicago truncatula. *Plant Cell* **26,** 1818–1830 (2014).

37. Zhang, X., Pumplin, N., Ivanov, S. & Harrison, M. J. EXO70I Is Required for Development of a Sub-domain of the Periarbuscular Membrane during Arbuscular Mycorrhizal Symbiosis. *Curr. Biol.* **25,** 2189–95 (2015).

38. Wang, E. *et al.* A common signaling process that promotes mycorrhizal and oomycete colonization of plants. *Curr. Biol.* **22,** 2242–6 (2012).

39. Javot, H., Penmetsa, R. V., Terzaghi, N., Cook, D. R. & Harrison, M. J. A Medicago truncatula phosphate transporter indispensable for the arbuscular mycorrhizal symbiosis. *Proc. Natl. Acad. Sci. U. S. A.* **104,** 1720–5 (2007).

40. Zhang, Q., Blaylock, L. a & Harrison, M. J. Two Medicago truncatula half-ABC transporters are essential for arbuscule development in arbuscular mycorrhizal symbiosis. *Plant Cell* **22,** 1483–97 (2010).

41. Gobbato, E. *et al.* A GRAS-type transcription factor with a specific function in mycorrhizal signaling. *Curr. Biol.* **22,** 2236–41 (2012).

42. Devers, E. a, Teply, J., Reinert, A., Gaude, N. & Krajinski, F. An endogenous artificial microRNA system for unraveling the function of root endosymbioses related genes in Medicago truncatula. *BMC Plant Biol.* **13,** 82 (2013).

43. Xue, L. *et al.* Network of GRAS Transcription Factors Involved in the Control of Arbuscule Development in. *Plant Physiol.* **167,** 1–38 (2015).

44. Yu, N. *et al.* A DELLA protein complex controls the arbuscular mycorrhizal symbiosis in plants. *Cell Res.* **24,** 130–3 (2014).

45. Bravo, A., York, T., Pumplin, N., Mueller, L. A. & Harrison, M. J. Genes conserved for arbuscular mycorrhizal symbiosis identified through phylogenomics. *Nat. Plants* **2,** 15208 (2016).
